# Shared and vaccine-specific factors associated with COVID-19 and influenza vaccination intentions among adults in Poland: a COM-B-informed cross-sectional study

**DOI:** 10.3389/fpubh.2026.1961701

**Published:** 2026-10-06

**Authors:** Mariusz Duplaga, Magdalena Sikorska, Kinga Kowalska-Duplaga

**Affiliations:** 1 Department of Health Promotion and e-Health, Faculty of Health Sciences, Institute of Public Health, Jagiellonian University Medical College, Krakow, Poland; 2 Department of Pediatrics, Gastroenterology and Nutrition, Medical Faculty, Jagiellonian University Medical College, Krakow, Poland

**Keywords:** COM-B model, COVID-19 vaccination, health literacy, influenza vaccination, vaccination intention, vaccine hesitancy

## Abstract

**Introduction:**

Vaccination intentions reflect both vaccine-specific attitudes and cognitive, social, and practical conditions. This study aimed to identify shared and vaccine-specific factors associated with COVID-19 and influenza vaccination intentions among adults in Poland using a Capability, Opportunity, Motivation–Behavior (COM-B) model-informed comparative approach and to assess the incremental contribution of vaccine hesitancy.

**Methods:**

A cross-sectional computer-assisted web survey was conducted in May 2026 among 3,000 adults recruited from a national online panel using quota controls for age, sex, education, place of residence, and macroregion. Measures included a COM-B-informed item set, the Polish Adult Vaccine Hesitancy Scale, the short version of European Health Literacy Survey Questionnaire, the Conspiracy Mentality Questionnaire, and sociodemographic, health-related, political, religious, and social media variables. The item set was evaluated through exploratory and confirmatory factor analyses in independent subsamples, followed by theory-ordered hierarchical logistic regression, *post hoc*, and sensitivity analyses.

**Results:**

Intention was reported by 18.2% of respondents for COVID-19 vaccination and 31.5% for influenza vaccination. Psychometric analyses supported retention of three theory-defined COM-B domain indicators, although Capability and Opportunity showed limited empirical separation. Capability was positively associated with COVID-19 and influenza vaccination intentions (OR = 1.31 and 1.74, respectively), as was Motivation (OR = 2.64 and 1.56). Opportunity showed inverse adjusted associations (OR = 0.72 and 0.73). Vaccine hesitancy added explanatory value in both models and showed a nonlinear association with influenza intention. Political preference showed a vaccine-specific pattern: compared with supporters of Law and Justice, supporters of the far-right Confederation party had lower odds of COVID-19 vaccination intention (OR = 0.50), whereas no corresponding association was observed for influenza vaccination intention. Non-participation in the 2023 parliamentary election was associated with lower odds of both intentions. Nagelkerke *R*^2^ values were 0.387 and 0.392, and apparent discrimination was good (AUC = 0.857 and 0.833).

**Conclusion:**

The shared pattern across outcomes supports COM-B as an organizing framework, while the incremental contribution of hesitancy and differences in political and health-related correlates demonstrate the vaccine-specific context of vaccination intentions. COVID-19 intention remained more politically differentiated. Vaccination-promotion strategies should combine behavioral diagnosis with attention to the political context of each vaccine.

## 1 Introduction

Vaccination intention refers to a person’s stated readiness to receive a vaccine before that decision is translated into action. Although intention does not invariably lead to uptake, it remains relevant when vaccination is planned for the future, administered periodically, or not yet available at the time of assessment. The translation of intention into behavior depends on its strength and stability, the time remaining before vaccination, practical access, exposure to new information, and changes in personal circumstances (1, 2). Vaccination intention should therefore not be perceived merely as a temporary opinion. It represents an important stage in the decision-making process and is closely related to subsequent behavior. Assessing intention also helps identify the factors associated with readiness to vaccinate.

Vaccination decisions are vaccine- and context-specific (3). The same person may accept one vaccine while delaying or refusing another because many involved factors, including perceived disease risk, vaccine familiarity, recommendations, previous experience, and expectations concerning benefits and adverse effects may differ. Influenza vaccination is a recurring seasonal decision supported by long-standing recommendations and, for some adults, repeated personal experience. Decisions about further COVID-19 vaccination are shaped by a different history, including the exceptional circumstances of the pandemic, previous infection and vaccine doses, changing recommendations, and a decline in the perceived severity of the disease. According to available reviews influenza and COVID-19 vaccination intentions share determinants such as confidence in safety and effectiveness, perceived need, previous vaccination, social influence, and access, but the relative importance of these factors varies across vaccines and populations (4–6). The two intentions should therefore be examined as related but distinct outcomes rather than treated as interchangeable expressions of a general attitude toward vaccination.

Vaccination intentions may also vary with sociodemographic characteristics, health status, presence of chronic disease, and contact with healthcare services. These factors can reflect differences in medical need, access, prior experience, and exposure to professional recommendations, although their associations change across settings and vaccines (5–7). Age and chronic illness may increase the need for protection, while education and income may influence access to information and services. Contact with healthcare may be an opportunity for recommendation and discussion, but its relevance depends on the content and credibility of the interaction. Political and religious affiliations may additionally shape the legitimacy attributed to institutional sources. Personal characteristics and medical risk therefore provide an important background but do not fully explain for how people perceive a specific vaccine or form an intention to receive it.

Health literacy may contribute to vaccination decisions by supporting access to, understanding of, and appraisal of information about disease risk, vaccine benefits and safety, eligibility, and service access. It encompasses decision-making capacities in healthcare, disease prevention, and health promotion rather than factual knowledge alone (8). Higher health literacy may facilitate the comparison of competing claims and use of professional recommendations, but studies have reported heterogeneous associations with vaccination attitudes, intention, and uptake (7, 9). Health literacy should be considered within a broader decision-making context that also includes trust, prior beliefs, perceived risk, and opportunities to act.

Vaccination decisions also occur within social, cultural, and political contexts. Trust in healthcare professionals, public institutions, science, and vaccine manufacturers can shape responses to recommendations, while political identity and religious involvement may influence preferred sources of authority and interpretations of risk. Family members, peers, and community leaders may reinforce or weaken acceptance through norms and personal examples. These influences are particularly relevant when recommendations change or vaccination becomes publicly questioned. Although their direction varies across countries and periods, they form part of the environment in which vaccination information is interpreted and intentions are formed (3, 10).

Social media are an important component of this environment. They facilitate access to health information, interpersonal exchange, and public health communication, but their speed, reach, user-generated content, and limited editorial control also support the circulation of misleading claims. Vaccine-related misinformation is prominent online and may be reinforced within communities that repeatedly assume negative narratives (11, 12). Cross-national and review evidence has linked social media environments with vaccine hesitancy (13, 14). General social media use does not indicate which content a person encountered or trusted, but it may characterize the information environment in which vaccination decisions are formed.

Conspiracy mentality is a related but distinct construct: a generalized tendency to explain important events through concealed motives and coordinated wrongdoing by powerful groups. In vaccination contexts, conspiracy narratives commonly claim that authorities, experts, or manufacturers as concealing risks, exaggerating disease threats, or pursuing political or commercial interests. Available evidence has linked anti-vaccine conspiracy beliefs with lower vaccination intention and stronger anti-vaccination attitudes (15, 16). Polish research has similarly associated conspiracy beliefs with pandemic responses, COVID-19 vaccination uptake, susceptibility to health misinformation, and vaccine hesitancy (10, 17, 18). Their inclusion may capture a generalized distrustful orientation extending beyond conventional measures of vaccine attitudes.

Vaccination determinants have been organized in several frameworks. The 5C model distinguishes confidence, complacency, constraints, calculation, and collective responsibility, while the 7C model adds compliance and conspiracy (19, 20). These models provide vaccine-specific taxonomies of the psychological antecedents of vaccination readiness.

COM-B offers a broader behavioral framework in which Capability, Opportunity, and Motivation jointly shape behavior (21). Capability concerns the knowledge and skills needed to act, Opportunity the physical and social conditions that enable or constrain action, and Motivation the reflective and automatic processes that direct it. Applied to vaccination intentions, COM-B allows behavior-specific knowledge, practical access, social influence, and motivational processes to be examined within one structure. Its relationship to the 5C and 7C models is conceptual rather than one-to-one: many vaccine-specific antecedents fall primarily within Motivation, whereas constraints may reflect Capability, Opportunity, or motivational conflict. This broader organization supports comparison of COVID-19 and influenza vaccination intentions while retaining the distinct context of each decision. COM-B has previously been applied to vaccination willingness, intention, and uptake (22–24).

Vaccine hesitancy provides a perspective focused specifically on vaccination. It describes delay in acceptance or refusal despite the availability of vaccination services and is understood as a continuum encompassing doubts, postponement, selective acceptance, and vaccination despite concerns (3). The Vaccine Hesitancy Scale assesses confidence in recommendations, perceived importance and effectiveness, concerns about adverse events, and doubts about the need for vaccination (25). The Adult Vaccine Hesitancy Scale (AVHS) adapts this approach to adults’ own vaccination decisions, and the Polish version has demonstrated satisfactory reliability and validity (26, 27). Unlike COM-B domains specified in relation to a behavioral target, it captures a more general orientation toward adult vaccination.

Considering vaccine hesitancy together with COM-B distinguishes general vaccine attitudes from conditions related to a specific vaccination decision. A person may report adequate knowledge, access, and social support but still distrust routine recommendations or remain concerned about newer vaccines. Conversely, a generally hesitant person may intend to receive a particular vaccine when perceived need, professional recommendation, and practical opportunity align. The hesitancy measure also captures content related to confidence, complacency, and calculation in the 5C and 7C traditions. It does not reproduce either framework in full, but introduces a measure of general adult vaccine hesitancy alongside the COM-B domain indicators. Its incremental contribution can therefore indicate whether general vaccine hesitancy accounts for additional variation after capability, opportunity, and motivation related to the particular vaccination have been considered.

Adult vaccine hesitancy and conspiracy mentality represent related but conceptually distinct constructs. Vaccine hesitancy refers specifically to doubts, concerns, and evaluations regarding vaccination, whereas conspiracy mentality reflects a broader tendency to interpret important events as the result of secret actions by powerful groups. Although conspiracy-related beliefs are incorporated into some multidimensional models of vaccination readiness, including the 7C framework, a general conspiracy mentality is not limited to vaccine-specific narratives and may also shape trust in institutions, experts, and public authorities. The two constructs should therefore not be treated as interchangeable, as they may capture different cognitive and attitudinal correlates of vaccination intention.

Previous studies have compared COVID-19 and influenza vaccination intentions within a single study, focusing on theory-based psychological constructs, behavioral immune-system variables, or reasoned-action predictors (28–30). However, these studies did not combine COM-B domains, adult vaccine hesitancy, and determinants from the wider social, political, and information environment. Such comparisons can indicate which factors are common to vaccination decisions and which are more closely associated with the history, perceived need, or social meaning of each vaccine. This is particularly relevant in Poland, where vaccination has been associated with conspiracy beliefs, political and social context, and susceptibility to health misinformation (10, 18).

This study aimed to identify shared and vaccine-specific factors associated with intentions to receive COVID-19 and influenza vaccines among adults in Poland. Using a theory-driven comparative approach, we examined the Capability, Opportunity, and Motivation domains of COM-B together with adult vaccine hesitancy, sociodemographic and health-related characteristics, healthcare use, health literacy, political and religious context, social media use, and conspiracy mentality. The study also evaluated the psychometric performance of the COM-B-informed item set in independent exploratory and confirmatory subsamples and assessed whether adult vaccine hesitancy contributed additional information beyond the three COM-B domain indicators. Additional *post hoc* and sensitivity analyses were used to examine the interpretation and robustness of the main findings.

## 2 Materials and methods

### 2.1 Study design, setting, and participants

Data were collected between May 20 and May 26, 2026, using a computer-assisted web interviewing survey conducted by IQS Sp. z o.o. (31) through its online research panel. Eligible participants were members of the IQS online research panel who were aged 18–75 years, resided in Poland, and provided electronic informed consent to participate. Recruitment was conducted using prespecified quota controls for age, sex, educational attainment, place of residence, and NUTS 1 macroregion to approximate the distribution of the target adult population. NUTS 1 macroregion was used as a recruitment quota variable to ensure broad geographic coverage and was not included as a predictor in the regression models. Because participants were recruited from an opt-in panel and individual selection probabilities were unknown, the study used a non-probability quota sample. All descriptive and inferential analyses were conducted using unweighted data.

Individuals were excluded if they did not provide consent, did not meet the eligibility criteria, were unable to continue for technical reasons, submitted an incomplete questionnaire, or failed the prespecified data-quality checks. A total of 12,432 invitations were sent to members of the panel. Of those invited, 4,074 entered the questionnaire after nonparticipants, individuals who declined consent, and those unable to continue for technical reasons were excluded. Following data-quality assessment, 1,074 cases were excluded. These included 290 incomplete questionnaires and 784 completed questionnaires that failed the attention checks, were completed in less than one-third of the median completion time, or showed straight-line response patterns across a substantial part of the questionnaire. The final dataset comprised 3,000 completed and approved interviews.

A formal *a priori* power calculation was not performed. The target sample size of 3,000 was determined pragmatically to provide sufficient observations for the planned split-sample psychometric evaluation, including independent EFA and CFA subsamples of 1,500 participants each, hierarchical multivariable logistic regression models, and the assessment of less prevalent response categories and the tails of the measured attitude distributions. Subsamples of 1,500 respondents each were considered adequate for the planned analyses of a short item set with up to three factors, while allowing the exploratory findings to be evaluated in an independent confirmatory sample. Under simple random sampling assumptions, a sample of 3,000 corresponds to a maximum 95% margin of error of approximately ±1.8 percentage points for an estimated proportion of 50%. Because participants were recruited from a non-probability quota panel, this value is provided only as a nominal precision benchmark and should not be interpreted as a design-based sampling error or evidence of population representativeness.

The study was conducted in accordance with the Declaration of Helsinki and was approved by the Research Ethics Committee of Jagiellonian University Medical College (Decision No. 118.0043.1.336.2025; July 23, 2025). Participation was voluntary, and informed consent was obtained electronically before respondents entered the survey. No identifying information was included in the dataset provided to the research team, and survey responses could not be linked by the researchers to individual participants. The study was reported in accordance with the Strengthening the Reporting of Observational Studies in Epidemiology (STROBE) statement for cross-sectional studies; the completed checklist is provided in Supplementary Table S20.

### 2.2 Questionnaire

The questionnaire contained 88 individual questions. They covered adult vaccination attitudes and history, future vaccination intentions, COM-B-informed statements, general health literacy, adult vaccine hesitancy, Internet and social media use, conspiracy mentality, health status, use of physician services, information sources, sociodemographic characteristics, and questions concerning vaccination of children. Only variables used in the present analyses are described below.

The COVID-19 and influenza intention questions and the COM-B-informed statements were developed for the present survey. Previously published instruments used in the current analyses were the 10-item Adult Vaccine Hesitancy Scale (AVHS) (26, 27), the six-item European Health Literacy Survey Questionnaire (HLS-EU-Q6) (32), and the five-item Conspiracy Mentality Questionnaire (CMQ) (33). The ordering of items within the health-literacy, vaccine-hesitancy, and conspiracy-mentality sections was rotated in the online questionnaire.

#### 2.2.1 Vaccination-intention outcomes

Respondents were asked, “Do you intend to be vaccinated in the future against:” followed by two vaccine-specific items: COVID-19 and influenza in the next autumn-winter season. Each item had two response options, yes and no. Separate binary dependent variables were created and coded 1 for yes and 0 for no. The outcomes were analyzed separately because they represented different vaccine-specific decisions.

#### 2.2.2 COM-B-informed items and domain indicators

Twelve statements were developed deductively using the definitions of Capability, Opportunity, and Motivation in the COM-B framework (21) (Supplementary Table S1). The items were written specifically for adult vaccination and were informed by the range of vaccination beliefs and enabling conditions considered in previous COM-B-framed vaccination research (22). They were not a translation or adaptation of an established COM-B vaccination scale. The item set was organized according to relevant content coverage; numerical equality across domains was not treated as a requirement because COM-B specifies functional components of behavior rather than parallel psychometric subscales.

Capability items addressed knowledge of vaccination schedules and recommendations, perceived ability to recognize false information about vaccination, knowledge of where vaccination was available, and understanding of the difference between mandatory and recommended vaccination. Opportunity items addressed perceived local access, the possibility of consulting a physician about vaccination, and encouragement to vaccinate at work or school. Motivation items addressed the views of close others, perceived public-health benefits of vaccination, willingness to vaccinate to protect others, trust in institutions recommending vaccination, and concern about post-vaccination complications. Responses ranged from 1 (strongly disagree) to 5 (strongly agree).

The initial 12-item set was evaluated using exploratory and confirmatory factor analyses in two independent random subsamples of 1,500 respondents each. The detailed extraction, estimation, model-comparison, and fit criteria are reported in the Statistical analysis subsection. The full wording of the COM-B Items and final retention decisions are provided in Supplementary Table S1.

#### 2.2.3 Adult vaccine hesitancy scale

General adult vaccine hesitancy was assessed with the 10-item Polish Adult Vaccine Hesitancy Scale (AVHS) (26, 27). The instrument assesses respondents’ own vaccination attitudes and was derived from the Vaccine Hesitancy Scale originally developed for parental vaccination decisions (25). The scale was administered once, without separate versions for COVID-19 and influenza, and the same AVHS score was used in both vaccination-intention models. Items were rated on a five-point agreement scale. Responses were recoded so that higher values consistently represented greater vaccine hesitancy; items 5, 9, and 10 required reverse orientation relative to the remaining items. The present analyses used the mean of all 10 items, with a possible range from 1 to 5. The AVHS showed high internal consistency in the full sample (Cronbach’s *α* = 0.919; McDonald’s *ω* = 0.919).

#### 2.2.4 Health literacy

General health literacy was measured using the six-item European Health Literacy Survey Questionnaire (HLS-EU-Q6), a brief version of the HLS-EU conceptual and measurement framework (32). The items ask how difficult respondents find selected tasks involving access to, understanding of, appraisal of, and use of health information. Responses were coded from 1 (impossible) to 4 (very easy). “Difficult to say/not applicable” was treated as a nonvalid response for score calculation. A mean score was calculated when at least five items had valid responses. Scores of 2 or lower indicated inadequate health literacy, scores above 2 and up to 3 indicated problematic health literacy, and scores above 3 indicated sufficient health literacy. For regression analysis, inadequate and problematic health literacy were combined, sufficient health literacy formed the second category, and respondents without enough valid responses were retained in an undetermined category. The HLS-EU-Q6 showed acceptable internal consistency in the present sample (Cronbach’s *α* = 0.787; McDonald’s *ω* = 0.786).

#### 2.2.5 Conspiracy mentality

Conspiracy mentality was assessed using the five-item Conspiracy Mentality Questionnaire based on the instrument developed by Bruder et al. (33, 34). The items refer to beliefs that governments conceal important information from the public, secret organizations influence political decisions and other aspects of everyday life, apparently unrelated events are connected through covert activities, and citizens are monitored without their knowledge. Respondents rated the likelihood that each statement was true on a scale ranging from 0% (“certainly not”) to 100% (“certain”), in 10-percentage-point increments. Responses were coded from 0 to 100, with higher values indicating stronger endorsement of each statement. The overall score was calculated as the mean of the five items, with higher scores indicating a stronger conspiracy mentality. The questionnaire demonstrated high internal consistency in the present sample (Cronbach’s *α* = 0.882; McDonald’s *ω* = 0.888).

#### 2.2.6 Daily social media use

Daily social media use was assessed with one question asking how much time respondents usually spent on platforms such as Facebook, Twitter/X, or Instagram. For the regression analyses, responses were grouped as no use, less than 30 min, 30–60 min, 61–120 min, and more than 120 min per day. This variable was treated as a measure of time spent using social media, not as a measure of exposure to vaccine misinformation or problematic social media use.

#### 2.2.7 Health status and use of physician services

Self-rated health was assessed using five response options ranging from excellent to unsatisfactory and was grouped for analysis as unsatisfactory, satisfactory, good, and very good or excellent. Respondents were asked whether they had a disease lasting more than 6 months, with response options “yes,” “no,” and “difficult to say.” For regression analysis, a binary indicator distinguished who reported a chronic disease (“yes”) from those who did not provide a definite affirmative response (“no” or “difficult to say”). The “difficult to say” option was selected by 229 participants (7.6%).

Respondents were also asked how many times during the previous 2 years they had visited a primary care/family physician or a medical specialist in either public or private care, with the two types of visit reported together. Response categories were no visits, once, two to three times, four to five times, and more than five times. The original categories were retained in the regression models, with no visits as the reference category.

#### 2.2.8 Sociodemographic and worldview-related variables

The sociodemographic variables included age in years, sex, place of residence, educational attainment, marital status, and monthly net household income per household member. Place of residence was categorized as rural area; town below 20,000 inhabitants; town or city with 20,001–100,000, 100,001–200,000, or 200,001–500,000 inhabitants; and city with more than 500,000 inhabitants. Education was grouped as lower than secondary, secondary, post-secondary non-university, bachelor’s degree, and master’s degree or equivalent. Marital status was grouped as single, married, widowed, divorced or separated, and living in a partnership. Income was grouped as PLN 0–2,000, PLN 2,001–3,000, PLN 3,001–5,000, above PLN 5,000, and refusal to report income.

Political preference was assessed by asking which electoral committee or party the respondent supported in the 2023 election to the Sejm. The analytical categories were Law and Justice, Third Way, New Left, Confederation, Civic Coalition, other party/invalid vote/unable to vote, and non-participation in the election; Law and Justice was the reference category in the final regression coding. Frequency of religious practice was grouped as non-believer, believer but non-practicing, less than once per month, 1–3 times per month, and more than 3 times per month. Non-believers were the reference category in the regression models.

### 2.3 Statistical analysis

Analyses were conducted using IBM SPSS Statistics 31.0.1.0. Psychometric analyses were performed in R 4.4 embedded in SPSS using the psych 2.6.3, lavaan 0.7–2, and semTools 0.5–8 packages (35). Categorical variables were summarized as counts and percentages, and continuous variables as means and standard deviations. All statistical tests were two-sided, with *p* < 0.05 indicating statistical significance. Generative AI (ChatGPT, web version; GPT-5.6 model; OpenAI; accessed between July 21 and August 5, 2026) was used to assist with drafting and refining selected R code. The AI-assisted code was reviewed, tested, and executed by the authors through the R integration in IBM SPSS Statistics, and the resulting analyses were verified against the source data and the corresponding SPSS and R output.

#### 2.3.1 Psychometric evaluation of the COM-B-informed item set

The initial investigator-developed, COM-B-informed item set comprised 12 statements rated from 1 to 5. COM-B Item 12 was reverse-scored (Item 12_R = 6 − Item 12), so that higher scores consistently represented conditions more favorable to vaccination. The full sample was randomly divided into two non-overlapping subsamples by generating a uniformly distributed random variable in IBM SPSS Statistics, sorting the cases according to its value, and assigning the first 1,500 cases to the EFA subsample and the remaining 1,500 cases to the CFA subsample.

EFA was conducted using polychoric correlations, given the ordinal response format (36). The number of factors was evaluated using parallel analysis with 200 permuted datasets, together with the interpretability and stability of competing solutions (37, 38). One-, two-, and three-factor solutions were estimated using minimum residual extraction and oblimin rotation. COM-B Item retention was based on loading patterns, communalities, and substantive consistency with the COM-B domains. COM-B Item 7 was removed because it had no salient loading and very low communality, after which the two- and three-factor solutions were re-estimated. Detailed item diagnostics, parallel analysis, and EFA results are reported in Supplementary Tables S2–S8.

CFA in the independent subsample compared an 11-item one-factor model, a two-factor model combining Capability and Opportunity while retaining Motivation as a separate factor, and the theoretically specified three-factor model comprising Capability (COM-B Items 1–4), Opportunity (COM-B Items 5–6), and Motivation (COM-B Items 8–11 and reverse-scored COM-B Item 12). Ordered indicators were analyzed using WLSMV estimation with theta parameterization (39, 40). Model fit was assessed using scaled *χ*^2^, CFI, TLI, RMSEA with its 90% confidence interval, and SRMR, and the two- and three-factor models were compared using a scaled *χ*^2^ difference test appropriate for WLSMV (41). CFA model fit, standardized loadings, reliability, and discriminant-validity results are presented in Supplementary Tables S9–S13.

Model-based omega was calculated for each domain in the CFA sample (42), together with average variance extracted (43). Discriminant validity was evaluated using the Fornell–Larcker criterion (43), HTMT2 computed from polychoric correlations (44, 45), and latent-factor correlations (46). The three-factor structure with 11 retained items was used to construct the COM-B domain indicators for the regression analyses. Three unweighted domain indicators were subsequently calculated on the original 1–5 scale: Capability as the mean of COM-B Item 1–4, Opportunity as the mean of COM-B Item 5–6, and Motivation as the mean of COM-B Item 8–11 and reversed COM-B Item 12.

#### 2.3.2 Hierarchical logistic regression models

Separate hierarchical binary logistic regression models were estimated for COVID-19 and influenza vaccination intentions, coded as 1 for the presence and 0 for the absence of intention. Predictors were entered by forced entry in four theoretically ordered blocks. Block 1 comprised age, sex, place of residence, educational attainment, marital status, household income, self-rated health, chronic disease status, use of physician services in the previous 2 years, and health-literacy category. Block 2 added political preference, frequency of religious practice, daily social media use, and conspiracy mentality. Block 3 added the Capability, Opportunity, and Motivation indicators. Block 4 added the mean AVHS score to evaluate its incremental contribution beyond the preceding blocks.

The AVHS mean score was centered by subtracting the sample mean from each participant’s score before its inclusion in the regression models. The Box–Tidwell test with Bonferroni correction indicated a departure from linearity in the logit for the AVHS score in the influenza model. Therefore, the centered linear term and the square of the centered term were entered jointly in the final influenza model. The COVID-19 model included only the centered linear term because no evidence of nonlinearity was found for this outcome.

Before model estimation, variable coding, missingness, outcome-by-predictor cell counts, and model convergence were examined. Multicollinearity was assessed using tolerance and variance inflation factors. For this diagnostic, the auxiliary linear model included the uncentered AVHS mean score and did not include the squared term used in the final influenza model. Linearity in the logit was evaluated for continuous predictors using Box–Tidwell terms with Bonferroni correction (47). Studentized and deviance residuals, Cook’s distance, and leverage were examined to identify potentially influential observations. These diagnostics did not lead to the exclusion of any observations. Model specification and diagnostic assessment followed standard recommendations for logistic regression (48, 49). A consolidated summary of diagnostics and final-model performance is provided in Supplementary Table S14.

Categorical predictors were represented by indicator variables using the reference categories shown in the regression tables. Improvement after each block was assessed using the likelihood-ratio *χ*^2^ test. Model performance was summarized using −2 log likelihood, Cox–Snell *R*^2^, Nagelkerke *R*^2^, the Hosmer–Lemeshow test, and the area under the receiver operating characteristic curve with its 95% confidence interval. Associations are reported as odds ratios with 95% confidence intervals.

#### 2.3.3 *Post hoc* and sensitivity analyses

To examine the sign reversal of the Opportunity coefficient, seven *post hoc* specifications were estimated separately for each vaccination outcome: Opportunity alone; Opportunity with the Block 1 and 2 covariates; Opportunity with Capability; Opportunity with Motivation; Opportunity with Capability and Motivation; the complete model containing Blocks 1 and 2 and all three COM-B indicators before vaccine hesitancy; and the final model including vaccine hesitancy. For influenza intention, the final specification included both the centered linear and squared AVHS terms. Changes in the Opportunity coefficient, odds ratio, and *p* value were compared across specifications; 95% confidence intervals were reported for the final estimates (Supplementary Table S15).

Because COM-B Item 10 referred to becoming vaccinated to protect others and therefore partially overlapped conceptually with the vaccination-intention outcomes, both hierarchical models were re-estimated using an alternative Motivation indicator comprising COM-B Items 8, 9, and 11 and reverse-scored COM-B Item 12. All other model specifications remained unchanged. Incremental fit, final-model fit, and key coefficient comparisons are reported in Supplementary Tables S16–S19.

## 3 Results

### 3.1 Sample characteristics

The study sample comprised 3,000 adults with a mean age of 44.63 years (SD = 14.40); 51.2% were women, 37.5% lived in rural areas, 49.8% were married, and 40.6% reported a chronic disease. Secondary education was the most frequent educational category (31.7%), followed by a master’s degree or equivalent (27.9%). Sufficient health literacy was identified in 60.4% of respondents, whereas 23.3% had inadequate or problematic health literacy and 16.3% had an undetermined score. Intention to receive an influenza vaccine was reported by 31.5% of respondents, compared with 18.2% for COVID-19 vaccination. The mean scores were 2.65 (SD = 0.78) for vaccine hesitancy and 64.81 (SD = 22.86) for conspiracy mentality. The COM-B domain means were 3.63 (SD = 0.71) for Capability, 3.95 (SD = 0.75) for Opportunity, and 3.26 (SD = 0.89) for Motivation (Table 1).

**Table 1:** Categorical characteristics of the study sample and vaccination intentions (*N* = 3,000).

Variable	Category	%	*n*
Sex	Female	51.2	1,537
	Male	48.8	1,463
Place of residence	Rural area	37.5	1,126
	Urban below 20,000 inhabitants	11.8	354
	Urban 20,001–100,000 inhabitants	22.0	661
	Urban 100,001–200,000 inhabitants	7.5	226
	Urban 200,001–500,000 inhabitants	7.6	229
	Urban more than 500,000 inhabitants	13.5	404
Educational attainment	Lower secondary or basic vocational	17.3	520
	Secondary	31.7	950
	Postsecondary non-university	11.4	341
	Bachelor’s or engineering degree	11.8	353
	Master’s degree or equivalent	27.9	836
Marital status	Single	24.5	736
	Married	49.8	1,493
	Widowed	3.2	97
	Divorced or separated	7.9	236
	Living in a partnership	14.6	438
Net monthly household income per person	PLN 0–2,000	12.7	380
	PLN 2,001–3,000	14.5	434
	PLN 3,001–5,000	31.2	935
	Above PLN 5,000	27.3	819
	Refused to report income	14.4	432
Political preference	Law and justice	19.6	589
	Third way	7.3	220
	New left	9.2	275
	Confederation	11.5	344
	Civic coalition	30.0	901
	Other parties, invalid vote, or unable to vote	5.5	165
	Did not participate in the election	16.9	506
Religious practice	Non-believer	18.5	554
	Believer, non-practicing	34.7	1,040
	Less than once per month	14.1	422
	1–3 times per month	12.7	381
	More than 3 times per month	20.1	603
Daily social media use	No use	9.9	296
	Less than 30 min per day	29.3	878
	30–60 min per day	23.2	695
	61–120 min per day	22.7	680
	More than 120 min per day	15.0	451
Self-rated health	Unsatisfactory	9.2	275
	Satisfactory	20.8	625
	Good	44.8	1,343
	Very good or excellent	25.2	757
Chronic disease	No or difficult to say	59.4	1,783
	Yes	40.6	1,217
Use of physician services in the previous 2 years	No use	10.0	300
	Once	12.1	364
	2–3 times	29.2	877
	4–5 times	18.7	560
	More than 5 times	30.0	899
Health literacy	Inadequate or problematic	23.3	698
	Sufficient	60.4	1,812
	Undetermined	16.3	490
COVID-19 vaccination intention	No	81.8	2,453
	Yes	18.2	547
Influenza vaccination intention	No	68.5	2,056
	Yes	31.5	944

### 3.2 Psychometric evaluation of the COM-B-informed item set

The EFA subsample contained no missing item responses, and all items used the full 1–5 response range. The correlation matrix was suitable for factor analysis (KMO = 0.879; Bartlett’s *χ*^2^(66) = 9,891.33, *p* < 0.001). Parallel analysis favored a two-factor solution: only the first two observed eigenvalues (5.351 and 1.826) exceeded the corresponding random 95th percentiles (Supplementary Tables S2, S3).

In the 12-item EFA, the two-factor solution separated a combined Capability–Opportunity factor from Motivation, whereas the three-factor solution more closely approximated the theoretically specified domains. COM-B Item 7 performed poorly in both solutions (largest loading ≤ 0.272; communality ≤ 0.081) and was removed. After its removal, the two-factor structure remained stable, while the three-factor solution had lower residuals (RMSR = 0.020; maximum absolute residual = 0.047). COM-B Item 3 continued to cross-load on Capability (0.517) and Opportunity (0.398) but was retained within Capability because its primary loading and content were consistent with that domain (Supplementary Tables S1, S4). Detailed EFA loadings and communalities are provided in Supplementary Tables S5–S8.

In the independent CFA subsample, the one-factor model fitted poorly, *χ*^2^(44) = 3,359.48, *p* < 0.001, CFI = 0.871, TLI = 0.839, RMSEA = 0.224 (90% CI 0.218–0.231), and SRMR = 0.153. Fit improved substantially for the two-factor model, *χ*^2^(43) = 619.15, *p* < 0.001, CFI = 0.978, TLI = 0.971, RMSEA = 0.095 (90% CI 0.088–0.101), and SRMR = 0.053. The three-factor model showed the best relative fit, *χ*^2^(41) = 523.23, *p* < 0.001, CFI = 0.981, TLI = 0.975, RMSEA = 0.089 (90% CI 0.082–0.095), and SRMR = 0.048, and fitted significantly better than the two-factor model, scaled Δ*χ*^2^(2) = 76.05, *p* < 0.001. Although the incremental indices and SRMR were favorable, RMSEA remained elevated. All models converged and showed no negative variance estimates (Supplementary Tables S9, S10).

In the three-factor model, standardized loadings ranged from 0.549 to 0.861 for Capability, 0.821 to 0.822 for Opportunity, and 0.649 to 0.907 for Motivation. Omega was 0.717, 0.730, and 0.889, respectively, and AVE was 0.580, 0.675, and 0.749. The latent Capability–Opportunity correlation was high (*r* = 0.840, 95% CI 0.815–0.865), whereas the Capability–Motivation and Opportunity–Motivation correlations were moderate (*r* = 0.505 and 0.445, respectively). Evidence for discriminant validity between Capability and Opportunity was mixed: the Fornell–Larcker criterion was not met, although HTMT2 was 0.789; HTMT2 values for Capability–Motivation and Opportunity–Motivation were 0.489 and 0.405. Taken together, the results provided qualified support for retaining the three theoretically defined domain indicators in the regression analyses, while showing limited empirical separation between Capability and Opportunity (Supplementary Tables S11–S13).

### 3.3 Hierarchical logistic regression models

#### 3.3.1 Model diagnostics and incremental fit

Both analyses included all 3,000 participants and had complete outcome and predictor data. COVID-19 vaccination intention was reported by 547 participants (18.2%) and influenza vaccination intention by 944 participants (31.5%). With 48 and 49 estimated predictor parameters, respectively, this corresponded to approximately 11.4 and 19.3 participants reporting vaccination intention per parameter. Cross-tabulations showed no empty or critically sparse outcome-by-predictor cells; the minimum outcome-by-political-preference cell counts were 19 for COVID-19 and 34 for influenza, and the smallest category of any predictor contained 97 participants.

Tolerance values ranged from 0.246 to 0.867 and VIF values from 1.153 to 4.071. No continuous predictor violated linearity in the logit in the COVID-19 model. In the influenza model, the Box–Tidwell term was significant only for vaccine hesitancy (*p* = 0.000175, Bonferroni-adjusted threshold *p* < 0.0083); adding the squared centered term improved fit beyond the linear term, Δ*χ*^2^(1) = 13.19, *p* < 0.001. Both final models converged after six iterations. Residual, leverage, and Cook’s-distance diagnostics did not identify an observation requiring exclusion; one influenza case had Cook’s distance = 0.816 but very low leverage (0.00124) (Supplementary Table S14).

All four prespecified predictor blocks significantly improved both models (Table 2). The largest increases in Nagelkerke *R*^2^ occurred after the COM-B indicators were entered in Block 3.

**Table 2:** Incremental fit of the hierarchical logistic regression models.

Outcome	Block	Δ*χ*^2^ (df)	*p* value	Nagelkerke *R*^2^	Δ*R*^2^
COVID-19	1	244.04 (29)	<0.001	0.127	–
	2	191.34 (15)	<0.001	0.220	0.093
	3	339.19 (3)	<0.001	0.371	0.151
	4	37.32 (1)	<0.001	0.387	0.016
Influenza	1	257.00 (29)	<0.001	0.115	–
	2	186.67 (15)	<0.001	0.193	0.078
	3	428.84 (3)	<0.001	0.354	0.161
	4	108.70 (2)	<0.001	0.392	0.038

Block 1: sociodemographic, health-related, use of physician services and health-literacy variables; Block 2: political preference, religious practice, social media use and conspiracy mentality; Block 3: COM-B Capability, Opportunity and Motivation; Block 4: mean-centered vaccine hesitancy for COVID-19 and mean-centered linear plus squared vaccine hesitancy terms for influenza. Δ*R*^2^ denotes the change in Nagelkerke *R*^2^ from the preceding block.

#### 3.3.2 COVID-19 vaccination intention

Nagelkerke *R*^2^ increased from 0.127 in Block 1 to 0.220 after addition of the sociopolitical and information-context variables, Δ*χ*^2^(15) = 191.34, *p* < 0.001. Entry of the COM-B indicators increased Nagelkerke *R*^2^ to 0.371, Δ*χ*^2^(3) = 339.19, *p* < 0.001, and vaccine hesitancy increased it to 0.387, Δ*χ*^2^(1) = 37.32, *p* < 0.001. The final model was significant, *χ*^2^(48) = 811.88, *p* < 0.001, and the Hosmer–Lemeshow test did not indicate lack of fit, *χ*^2^(8) = 11.29, *p* = 0.186.

In the final model, Capability (OR = 1.31, 95% CI 1.05–1.63) and Motivation (OR = 2.64, 95% CI 1.99–3.51) were positively associated with intention, whereas Opportunity was inversely associated (OR = 0.72, 95% CI 0.59–0.88). Higher vaccine hesitancy was associated with lower odds of intention (OR = 0.38, 95% CI 0.28–0.52). Older age (OR = 1.03 per year, 95% CI 1.02–1.04, *p* < 0.001) and female sex (OR = 1.70, 95% CI 1.34–2.17, *p* < 0.001) were also associated with higher odds of COVID-19 vaccination intention. Married respondents had lower odds than single respondents (OR = 0.63, 95% CI 0.45–0.87, *p* = 0.005). Compared with supporters of Law and Justice, supporters of Confederation (OR = 0.50, 95% CI 0.28–0.88, *p* = 0.017) and respondents who did not participate in the 2023 parliamentary election (OR = 0.59, 95% CI 0.38–0.93, *p* = 0.021) had lower odds of intention. Respondents living in cities with more than 500,000 inhabitants had higher odds of COVID-19 vaccination intention than rural residents (OR = 1.52, 95% CI 1.08–2.14, *p* = 0.017). Use of physician services two to three times, four to five times, or more than five times during the previous 2 years was also associated with higher odds than no use (ORs = 2.08, 2.03, and 2.05, respectively). Complete adjusted estimates are presented in Table 3.

**Table 3:** Final model for COVID-19 vaccination intention.

Variable	Category	OR	95% CI	*p* value
Age (per year)		1.03	1.02–1.04	<0.001
Sex	Male^#^			
	Female	1.70	1.34–2.17	<0.001
Place of residence	Rural area^#^			
	Urban area below 20,000	1.09	0.74–1.62	0.657
	Urban area 20,001–100,000	1.16	0.85–1.59	0.342
	Urban area 100,001–200,000	1.37	0.88–2.13	0.162
	Urban area 200,001–500,000	1.33	0.86–2.07	0.200
	Urban area more than 500,000	1.52	1.08–2.14	0.017
Education	Lower than secondary education^#^			
	Secondary education	0.83	0.58–1.18	0.290
	Post-secondary non-university education	0.90	0.58–1.40	0.639
	Bachelor’s degree	0.70	0.45–1.11	0.127
	Master’s degree	0.82	0.57–1.18	0.286
Marital status	Single^#^			
	Married	0.63	0.45–0.87	0.005
	Widowed	0.65	0.33–1.29	0.219
	Divorced or separated	0.90	0.56–1.44	0.657
	In partnership	0.999	0.681–1.463	0.994
Net monthly income	PLN 0–2,000^#^			
	PLN 2,001–3,000	0.81	0.51–1.29	0.368
	PLN 3,001–5,000	0.91	0.61–1.36	0.633
	Above PLN 5,000	0.94	0.62–1.42	0.776
	Refused to report income	0.83	0.52–1.32	0.424
Self-rated health	Unsatisfactory^#^			
	Satisfactory	1.14	0.73–1.78	0.574
	Good	1.05	0.68–1.64	0.819
	Very good or perfect	0.77	0.46–1.30	0.324
Chronic disease	No^#^			
	Yes	0.90	0.69–1.16	0.409
Use of physician services in the previous 2 years	No use^#^			
	Once	1.72	0.95–3.10	0.072
	2–3 times	2.08	1.22–3.53	0.007
	4–5 times	2.03	1.17–3.51	0.012
	More than 5 times	2.05	1.19–3.53	0.010
Health literacy category	Inadequate or problematic^#^			
	Sufficient	1.12	0.85–1.47	0.427
	Undetermined	0.94	0.64–1.38	0.746
Political preference	Law and justice (PiS)^#^			
	third way	0.64	0.39–1.04	0.073
	New left	0.86	0.55–1.33	0.488
	Confederation	0.50	0.28–0.88	0.017
	Civic coalition	0.74	0.53–1.04	0.081
	Other parties, invalid vote, or unable to vote	0.66	0.35–1.23	0.193
	Did not participate in elections	0.59	0.38–0.93	0.021
Religious practice	Non-believer^#^			
	Believer, non-practicing	0.88	0.64–1.21	0.420
	Less than once per month	0.66	0.44–1.00	0.048
	1–3 times per month	1.22	0.81–1.84	0.353
	More than 3 times per month	0.86	0.59–1.25	0.415
Daily social media use	No use^#^			
	Less than 30 min per day	0.84	0.57–1.25	0.392
	30–60 min per day	1.12	0.74–1.70	0.589
	61–120 min per day	1.11	0.73–1.70	0.619
	More than 120 min per day	0.87	0.54–1.39	0.554
Conspiracy mentality		0.996	0.991–1.001	0.124
COM-B Capability		1.31	1.05–1.63	0.016
COM-B Opportunity		0.72	0.59–0.88	0.001
COM-B Motivation		2.64	1.99–3.51	<0.001
m-c AVHS		0.38	0.28–0.52	<0.001

^#^, reference category; m-c AVHS, mean-centered vaccine hesitancy score, COM-B Capability, score for Capability domain; COM-B Opportunity, score for Opportunity domain, COM-B Motivation, score for Motivation domain.

ORs are adjusted for all variables included in the final model. For categorical predictors, ORs, 95% CIs, and *p* values are reported only for nonreference categories. Omnibus *p* values for multicategory predictors are not shown. ORs and confidence intervals are shown to two decimal places, except for conspiracy mentality, for which three decimals are retained to avoid rounding the estimate to 1.00. For the COM-B Capability, COM-B Opportunity, COM-B Motivation scores, and mean-centered vaccine hesitancy score, the OR represents a one-point increase on the original score scale.

#### 3.3.3 Influenza vaccination intention

Nagelkerke *R*^2^ increased from 0.115 in Block 1 to 0.193 after Block 2 and to 0.354 after entry of the COM-B indicators. The centered linear and squared vaccine-hesitancy terms jointly improved the model in Block 4, Δ*χ*^2^(2) = 108.70, *p* < 0.001, yielding a final Nagelkerke *R*^2^ of 0.392. The final model was significant, *χ*^2^(49) = 981.20, *p* < 0.001, and the Hosmer–Lemeshow test did not indicate lack of fit, *χ*^2^(8) = 6.46, *p* = 0.596.

Capability (OR = 1.74, 95% CI 1.45–2.10) and Motivation (OR = 1.56, 95% CI 1.25–1.94) were positively associated with influenza vaccination intention, whereas Opportunity was inversely associated (OR = 0.73, 95% CI 0.62–0.86). The centered linear (*B* = −1.49, *p* < 0.001) and quadratic (*B* = −0.39, *p* < 0.001) vaccine-hesitancy coefficients were both negative. Evaluated jointly, the linear and quadratic vaccine-hesitancy terms indicated a decline in the model-implied probability of intention across the observed hesitancy range, with a steeper decline at higher hesitancy values. Older age (OR = 1.012 per year, 95% CI 1.003–1.021, *p* = 0.010) and female sex (OR = 1.57, 95% CI 1.28–1.92, *p* < 0.001) were associated with higher odds of influenza vaccination intention. Respondents living in urban areas with 20,001–100,000 inhabitants had higher odds of influenza vaccination intention than rural residents (OR = 1.31, 95% CI 1.01–1.69, *p* = 0.040). Very good or excellent self-rated health was associated with lower odds than unsatisfactory health (OR = 0.61, 95% CI 0.40–0.94, *p* = 0.023), whereas all categories of physician-service use were associated with higher odds than no use (ORs = 1.79–2.70). Respondents who did not participate in the 2023 parliamentary election also had lower odds than supporters of Law and Justice (OR = 0.62, 95% CI 0.44–0.88, *p* = 0.007). Complete adjusted estimates are presented in Table 4.

**Table 4:** Final model for influenza vaccination intention.

Variable	Category	OR	95% CI	*p* value
Age (per year)		1.012	1.003–1.021	0.010
Sex	Male^#^			
	Female	1.57	1.28–1.92	<0.001
Place of residence	Rural area^#^			
	Urban area below 20,000	1.14	0.83–1.57	0.420
	Urban area 20,001–100,000	1.31	1.01–1.69	0.040
	Urban area 100,001–200,000	1.23	0.85–1.79	0.267
	Urban area 200,001–500,000	1.36	0.94–1.98	0.102
	Urban area more than 500,000	1.35	1.00–1.82	0.053
Education	Lower than secondary education^#^			
	Secondary education	1.02	0.76–1.37	0.909
	Post-secondary non-university education	1.18	0.82–1.70	0.386
	Bachelor’s degree	1.004	0.691–1.458	0.984
	Master’s degree	1.07	0.78–1.45	0.689
Marital status	Single^#^			
	Married	0.94	0.72–1.22	0.619
	Widowed	0.95	0.53–1.71	0.854
	Divorced or separated	1.05	0.70–1.58	0.820
	In partnership	1.18	0.86–1.63	0.298
Net monthly income	PLN 0–2,000^#^			
	PLN 2,001–3,000	0.79	0.54–1.14	0.203
	PLN 3,001–5,000	0.98	0.71–1.36	0.917
	Above PLN 5,000	0.91	0.65–1.28	0.602
	Refused to report income	0.77	0.53–1.13	0.185
Self-rated health	Unsatisfactory^#^			
	Satisfactory	1.09	0.75–1.59	0.636
	Good	0.84	0.58–1.21	0.337
	Very good or excellent	0.61	0.40–0.94	0.023
Chronic disease	No^#^			
	Yes	1.06	0.85–1.32	0.616
Use of health services in the previous 2 years	No use^#^			
	Once	1.79	1.10–2.90	0.018
	2–3 times	2.17	1.41–3.35	<0.001
	4–5 times	2.70	1.72–4.25	<0.001
	More than 5 times	2.47	1.58–3.86	<0.001
Health literacy category	Inadequate or problematic^#^			
	Sufficient	1.28	1.02–1.61	0.034
	Undetermined	1.20	0.87–1.64	0.269
Political preference	Law and justice^#^			
	Third way	0.86	0.59–1.28	0.465
	New left	1.18	0.81–1.72	0.402
	Confederation	0.78	0.52–1.16	0.221
	Civic coalition	0.90	0.68–1.19	0.455
	Other parties, invalid vote, or unable to vote	0.68	0.42–1.12	0.130
	Did not participate in elections	0.62	0.44–0.88	0.007
Religious practice	Non-believer^#^			
	Believer, non-practicing	1.20	0.91–1.58	0.208
	Less than once per month	0.91	0.64–1.28	0.575
	1–3 times per month	1.61	1.13–2.30	0.008
	More than 3 times per month	1.13	0.82–1.57	0.460
Daily social media use	No use^#^			
	Less than 30 min per day	1.17	0.83–1.67	0.375
	30–60 min per day	1.39	0.96–2.01	0.079
	61–120 min per day	1.26	0.87–1.84	0.221
	More than 120 min per day	1.02	0.67–1.53	0.944
Conspiracy mentality		1.002	0.998–1.007	0.361
COM-B Capability		1.74	1.45–2.10	<0.001
COM-B Opportunity		0.73	0.62–0.86	<0.001
COM-B Motivation		1.56	1.25–1.94	<0.001
m-c AVHS		0.22	0.16–0.31	<0.001
sm-c AVHS		0.67	0.54–0.84	<0.001

^#^, eference category; m-c AVHS, mean-centered vaccine hesitancy score; sm-c AVHS, squared mean-centered vaccine hesitancy score; COM-B Capability, score for capability domain; COM-B Opportunity, score for opportunity domain; COM-B Motivation, score for motivation domain.

ORs are adjusted for all variables included in the final model. For categorical predictors, ORs, 95% CIs, and *p* values are reported only for nonreference categories. Omnibus *p* values for multicategory predictors are not shown. ORs and confidence intervals are shown to two decimal places, except for age and conspiracy mentality, for which three decimals are retained to avoid rounding the estimate to 1.00. For the COM-B Capability, COM-B Opportunity, and COM-B Motivation scores, the OR represents a one-point increase on the original score scale. Because vaccine hesitancy was modeled using mean-centered linear and squared terms, the exponentiated coefficients for these terms must be interpreted jointly and do not represent independent one-point effects.

#### 3.3.4 Discrimination of the final models

The final models showed good apparent discrimination in the estimation sample. The AUC was 0.857 (95% CI 0.842–0.873) for COVID-19 vaccination intention and 0.833 (95% CI 0.818–0.848) for influenza vaccination intention (Table 5).

**Table 5:** Receiver operating characteristic analysis of the final models.

Outcome	AUC	SE	95% CI	*p* value
COVID-19	0.857	0.008	0.842–0.873	<0.001
Influenza	0.833	0.008	0.818–0.848	<0.001

AUC values represent apparent discrimination in the estimation sample (*N* = 3,000).

#### 3.3.5 *Post hoc* analysis of the COM-B opportunity coefficient

Opportunity was positively associated with both outcomes when entered alone and after adjustment for Blocks 1 and 2. Its coefficient was attenuated after Capability was added and became inverse when all three COM-B indicators were entered simultaneously. The inverse coefficient was already present before vaccine hesitancy was added and was only modestly strengthened in the final models. The complete seven-model sequence is presented in Supplementary Table S15.

#### 3.3.6 Sensitivity analysis excluding item 10 of COM-B instrument

Excluding COM-B Item 10 from the Motivation indicator produced small reductions in final-model Nagelkerke *R*^2^ for COVID-19 vaccination intention (0.387 to 0.381) and influenza vaccination intention (0.392 to 0.388). The directions and statistical significance of Capability, Opportunity, Motivation, and vaccine hesitancy were unchanged. The Motivation association was attenuated but remained significant for COVID-19 (OR = 2.13, 95% CI 1.64–2.76, *p* < 0.001) and influenza (OR = 1.27, 95% CI 1.03–1.56, *p* = 0.023). Detailed sensitivity-model estimates are provided in Supplementary Tables S16–S19.

## 4 Discussion

### 4.1 Principal findings

In this survey of 3,000 adults in Poland, influenza vaccination intention was more frequent than COVID-19 vaccination intention, but the hierarchical models showed a broadly similar core pattern. Capability and Motivation were positively associated with both outcomes, Opportunity had an inverse coefficient after mutual adjustment, and the COM-B block substantially improved both models. General adult vaccine hesitancy added information beyond the three COM-B domain indicators in both models. For influenza, the association with hesitancy was nonlinear, with the predicted probability of intention decreasing more steeply at higher hesitancy values. Use of physician services was associated with both vaccination intentions, and place of residence showed category-level associations in both models. Significant category-level associations with self-rated health and health literacy were observed only in the influenza model.

The findings support COM-B as an organizing framework, while the three scores should be interpreted as theory-based domain indicators rather than unequivocally independent psychometric dimensions. Exploratory analyses more clearly separated Motivation from a combined Capability–Opportunity dimension, whereas the three-factor CFA fitted better than the two-factor alternative but retained a high Capability–Opportunity correlation.

### 4.2 COM-B domain indicators and vaccination intention

Capability remained positively associated with both vaccination intentions after adjustment for the other COM-B indicators, vaccine hesitancy, and background covariates. The indicator covered perceived knowledge of vaccination recommendations, recognition of false information, knowledge of where vaccination could be obtained, and understanding of mandatory versus recommended vaccines. It therefore represented perceived cognitive resources for interpreting recommendations and navigating vaccination decisions. This finding is consistent with psychological Capability in COM-B and with evidence linking vaccination knowledge, information appraisal, and understanding of recommendations to acceptance (7, 21, 50). Because these competencies were self-assessed, the score may also reflect confidence in one’s understanding and prior engagement with vaccination information.

Motivation was also positively associated with both outcomes. Its content included social approval, perceived public health and interpersonal benefits, institutional trust, concern about adverse consequences, and prosocial orientation. These elements correspond to motivational processes within COM-B and to established correlates of vaccination, including confidence, perceived necessity and benefit, trust, affective concerns, social norms, and collective responsibility (1, 5, 6, 19, 51). The association remained after adjustment for general adult vaccine hesitancy, indicating that Motivation contributed information beyond the attitudes captured by the AVHS. Similar vaccine- and outcome-specific contributions of COM-B domains have been reported in an Australian study of COVID-19 vaccination and antiviral uptake (24).

The inverse Opportunity coefficient requires more cautious interpretation. The indicator reflected perceived ease of access and the possibility of consulting a physician, both of which would ordinarily be expected to support vaccination. Opportunity was positively associated with both intentions before simultaneous adjustment for the other COM-B indicators. Its negative final coefficient therefore represents the variation remaining after shared variance with Capability, Motivation, vaccine hesitancy, and other covariates had been taken into account, rather than an overall association between greater access and lower intention.

A conditional, suppression-related interpretation is plausible because Capability and Opportunity were strongly correlated and partly overlapped in content. They were nevertheless retained separately because they represented distinct COM-B conditions, showed acceptable reliability, and the three-factor CFA fitted better than the model combining them. Given the narrow coverage of Opportunity and the concentration of responses toward the favorable end of the scale, mutual adjustment may have isolated residual variation that no longer represented the broader meaning of access or consultation. Sign reversal can occur when shared variance is partitioned among correlated predictors (52, 53). The recurrence of this coefficient in both models makes the pattern noteworthy, but its meaning remains limited by the measurement and model specification. Broader measures of physical and social Opportunity are needed to determine whether it extends beyond the present analysis. The full *post hoc* coefficient sequence is presented in Supplementary Table S15.

### 4.3 Vaccine hesitancy and its incremental contribution

The AVHS score was entered after the COM-B indicators to assess whether general adult vaccine hesitancy contributed information beyond the broader behavioral framework. COM-B describes conditions shaping the likelihood of behavior, whereas hesitancy concerns uncertainty, reluctance, delay, or refusal related to vaccination and may vary across vaccines, settings, and time (3, 21). The content of the AVHS overlaps with Motivation, but the scale measures general adult vaccine hesitancy rather than hesitancy toward a particular vaccine (25, 27).

Vaccine hesitancy improved both models, while Motivation remained positively associated with both outcomes. Their simultaneous inclusion allowed the association of each measure with vaccination intention to be assessed after adjustment for the other. The two measures partly overlapped, but collinearity diagnostics supported their retention in the same models. For influenza, the association with hesitancy was nonlinear: adjusted predicted probabilities decreased across the observed score range, with a steeper decline at higher values. This pattern did not identify a natural threshold or a discrete subgroup of highly hesitant respondents; it showed that a constant linear logit slope did not adequately represent the data.

### 4.4 Sociodemographic, political, and health-related context

Older age and female sex were positively associated with both intentions. Such demographic associations vary across populations, periods, and outcome definitions, and should be interpreted in the context of the current Polish sample (4, 7).

Educational attainment and household income were not independently associated with either vaccination intention. Place of residence showed category-level associations in both models. Compared with rural residents, respondents living in cities with more than 500,000 inhabitants had higher odds of COVID-19 vaccination intention, while those living in urban areas with 20,001–100,000 inhabitants had higher odds of influenza vaccination intention.

The socioeconomic findings were mixed, with significant associations for selected categories of place of residence but not for education or household income. The absence of significant associations with education and household income may partly reflect adjustment for Capability, Opportunity, use of physician services, political orientation, and vaccine hesitancy. The broad categories used to describe education, income, and residence did not directly capture transport difficulties, occupational constraints, local service availability, or financial barriers. Reviews report heterogeneous and context-dependent associations of education, income, and residential setting with vaccination intentions (4–7).

Use of physician services was associated with both vaccination intentions. Compared with respondents reporting no physician visits in the previous 2 years, those reporting two to three, four to five, or more than five visits had higher odds of both vaccination intentions. For influenza, significantly higher odds were also observed among respondents reporting one visit. In the influenza model, very good or excellent self-rated health was associated with lower odds of intention than unsatisfactory health, and sufficient health literacy was associated with higher odds than inadequate or problematic health literacy. These self-rated health and health-literacy contrasts were not significant in the COVID-19 model. The available measures could not distinguish the roles of professional recommendation, morbidity, or preventive orientation in the associations with physician-service use. Previous reviews have reported heterogeneous relationships between health literacy and vaccination (9, 54).

Religious practice showed category-specific associations in both models rather than a consistent gradient, suggesting differences between particular patterns of participation rather than a uniform increase or decrease in intention with frequency of practice. Political preference showed a clearer vaccine-specific pattern. Compared with supporters of Law and Justice, Confederation supporters had substantially lower odds of COVID-19 vaccination intention, but no corresponding difference was observed for influenza. This finding is consistent with the politicization of COVID-19 vaccination and with earlier Polish studies reporting particularly low acceptance or uptake among Confederation supporters, whose political representatives opposed pandemic-control measures and COVID-19 vaccination (18, 55, 56). Similar associations between far-right affiliation and COVID-19 vaccine hesitancy have been observed elsewhere in Europe (57, 58), within a broader literature linking political context and trust to vaccination attitudes (59).

Respondents who had not participated in the parliamentary election had lower intention in both models. Unlike the Confederation contrast, this pattern was not confined to COVID-19 and may reflect broader political or institutional disengagement, although the available variables could not identify the mechanism. Marital status also differed between the outcomes: married respondents had lower odds of COVID-19 vaccination intention than single respondents, whereas no corresponding association was present for influenza.

Daily social-media use was not independently associated with either vaccination intention, despite the expected relevance of online information environments to vaccination attitudes. Frequency of use, however, provides no information about the platform, exposure to vaccine-related content, the direction or credibility of that content, or active engagement with it. Similar levels of daily use may encompass exposure to misinformation, corrective information, pro-vaccination messages, and content unrelated to health. Vaccine hesitancy and trust-related Motivation may also capture more proximal evaluations through which online information becomes associated with intention. General conspiracy mentality was likewise not independently associated with either outcome. As a broad worldview measure, it differs from vaccine-specific conspiracy beliefs, which have been linked to vaccination intention through mistrust and negative vaccine evaluations (15, 16, 60–63). Polish evidence also associates susceptibility to health misinformation with vaccine hesitancy (10). The null results are therefore specific to generic media-use frequency and generalized conspiracy mentality rather than targeted exposure to, or endorsement of, vaccine-related narratives.

### 4.5 Comparison of the COVID-19 and influenza models

The common directions of the COM-B indicators and vaccine hesitancy support a shared behavioral framework, but differences in prevalence and contextual correlates favor vaccine-specific interpretation. Use of physician services was associated with both vaccination intentions. Influenza intention showed category-level associations with self-rated health and health literacy, together with a nonlinear hesitancy pattern, consistent with a recurrent seasonal decision embedded in routine clinical care (6, 64). COVID-19 intention was distinguished by the Confederation contrast and the association with marital status, reflecting a decision that remained connected with pandemic experience, changing recommendations, and political conflict. Education, household income, and daily social-media use were not independently associated with either outcome. Residence showed category-level associations in both models, while the contrast between sufficient and inadequate or problematic health literacy was significant only in the influenza model. Comparative research similarly identifies overlap in safety, effectiveness, trust, and perceived necessity, alongside vaccine-specific concerns and decision contexts (5, 65–67).

Perceived novelty, effectiveness, and safety of mRNA vaccines may also be relevant to Motivation and hesitancy. In a Polish sample surveyed in 2021, exclusive preference for mRNA vaccine products was positively associated with COVID-19 vaccination intention (68). Polish post-authorization research documented product-specific differences in self-reported short-term reactions among healthcare workers (69). Accumulated vaccination experience and the interpretation of post-marketing safety evidence may provide additional context for differences between COVID-19 and influenza vaccination decisions.

### 4.6 Implications for research and vaccination communication

The findings support behavioral diagnosis that combines general adult vaccine hesitancy with the cognitive, motivational, and practical conditions represented by COM-B. Communication should not treat reluctance as a knowledge deficit alone. Information about recommendations, benefits, safety, and availability may support Capability, but clinician recommendation, reminders, convenient delivery, and multicomponent approaches may be more effective when matched to the relevant barrier (1, 70).

Motivational and hesitancy components should be examined separately in formative research, including trust, perceived benefit, social norms, and adverse-effect concerns. The nonlinear influenza pattern also supports analysis across the full hesitancy range rather than arbitrary categorization. The inverse Opportunity coefficient provides no basis for deprioritizing access; future studies should distinguish perceived from objective access and measure cost, distance, appointment availability, opening hours, clinician recommendation, and workplace or community support more comprehensively. The limited and category-specific associations of broad sociodemographic variables also indicate that these characteristics alone may be insufficient for audience segmentation. Formative assessment should identify vaccine-specific concerns, actual information exposure, and concrete access barriers within demographic groups.

### 4.7 Strengths and limitations

Strengths include the separate analysis of two vaccine-specific outcomes in a large sample, independent exploratory and confirmatory psychometric subsamples, methods appropriate for ordinal items, hierarchical models covering a broad range of determinants, and extensive regression diagnostics. The nonlinear influenza hesitancy association was incorporated into the final model, and both models showed good apparent discrimination.

The cross-sectional design does not establish temporal ordering among COM-B conditions, hesitancy, and intention, and reciprocal relationships with information seeking or perceived knowledge are possible. The outcomes were stated intentions rather than subsequent vaccination. The questions offered only “yes” and “no” responses, without a “do not know” or “undecided” option. This format did not capture the strength or certainty of intention and did not allow undecided respondents to be identified separately. A “no” response should therefore not be interpreted as a definite refusal to vaccinate. Actual uptake may also depend on changing recommendations, access, competing priorities, and later experiences (2).

The survey-specific COM-B indicators should not be treated as established subscales. Exploratory analyses more clearly supported a combined Capability–Opportunity dimension, while the better-fitting three-factor CFA retained a high correlation between these domains. Opportunity contained two items and covered a limited range of physical and social conditions, which constrains interpretation of its inverse coefficient. All principal variables were also self-reported in one questionnaire and may share response tendencies. Capability reflected perceived rather than tested knowledge, and Opportunity reflected perceived rather than objectively measured access. Future work could combine self-report with knowledge tests, service-access data, clinician recommendation, and vaccination records. The limited or category-specific findings for education, income, residence, health literacy, and social-media use must also be considered in relation to measurement granularity: settlement size and household income were proxies rather than direct measures of local or financial barriers, health literacy was grouped into broad categories, and daily use did not characterize platforms or vaccine-related exposure.

The findings are specific to Poland in 2026, after the acute pandemic period and under a particular set of recommendations and public debates. COVID-19 vaccination was shaped by accumulated infection and vaccination experience, whereas influenza remained a recurrent seasonal decision embedded in routine care. The magnitude of the observed associations may therefore differ across seasons, policy settings, and populations (66). Finally, the models were estimated and evaluated in the same sample, so their AUC values describe apparent discrimination and may be optimistic. Some potentially relevant determinants, including detailed vaccination history, clinician recommendation, prior adverse experiences, vaccine-specific disease-risk perceptions, and objective local service characteristics, were not included. Vaccination-intention questions did not specify vaccine type, and preferences for particular products or beliefs about mRNA vaccines were not assessed. Although Motivation and the AVHS included trust- and safety-related content, they did not distinguish general concerns from those specific to mRNA vaccines. We therefore could not evaluate whether these beliefs or accumulated experience with particular vaccine products contributed to differences between COVID-19 and influenza vaccination intentions. Future research could examine these factors by assessing product-specific beliefs alongside detailed vaccination histories and respondents’ interpretation of post-marketing evidence. External validation would be required before predictive use in other populations.

## 5 Conclusion

COVID-19 and influenza vaccination intentions appear to share a common behavioral architecture, but should not be treated as interchangeable expressions of a general pro-vaccination disposition. COM-B provides a useful structure for identifying this shared architecture, while adult vaccine hesitancy identifies an additional evaluative layer not fully represented by the three domains. The inverse Opportunity coefficient also illustrates that COM-B domain estimates are relational and depend on the behavioral and attitudinal components modeled alongside them. Vaccination-promotion strategies should therefore combine a behavioral diagnosis with vaccine-specific calibration: greater attention to political and social meaning for COVID-19, and to perceived health need for influenza. The association with physician-service use was observed for both vaccines. This perspective is more informative than treating hesitancy or access as uniform barriers across vaccines.

## Data Availability

The datasets presented in this study can be found in online repositories. The names of the repository/repositories and accession number(s) can be found at: Zenodo repository: https://doi.org/10.5281/zenodo.21841697.
