# Supplementary material for: Shared and vaccine-specific factors associated with COVID-19 and influenza vaccination intentions among adults in Poland: a COM-B-informed cross-sectional study
Source: Front Public Health. 2026 Oct 6;14:1961701. doi: 10.3389/fpubh.2026.1961701 (PMC13680461; doi:10.3389/fpubh.2026.1961701)
Supplement: Supplementary file 1 [file Data_Sheet_1.PDF]

Supplementary Material for

"Shared and vaccine-specific factors associated with COVID-19 and influenza vaccination intentions among adults in Poland: a COM-B-informed cross-sectional study."

Psychometric evaluation of the COM-B-informed item set

The initial investigator-developed COM-B-informed item set comprised 12 statements rated from 1 to 5. COM-B Item 12 was reverse-scored (Item 12_R = 6 − Item 12), so that higher values consistently represented conditions more favorable to vaccination. The full sample (N = 3,000) was randomly divided into non-overlapping exploratory factor analysis (EFA) and confirmatory factor analysis (CFA) subsamples of 1,500 respondents each.

The polychoric matrix used for EFA was estimated in R by a two-step maximum-likelihood procedure. Category thresholds were derived from marginal distributions, and pairwise latent correlations were estimated from 5 x 5 contingency tables using deterministic Gauss-Legendre quadrature. Parallel analysis used 200 independent permutations of each item while preserving its marginal distribution; the polychoric matrix was re-estimated for every replicate.

One-, two-, and three-factor solutions were estimated from the polychoric matrix using minimum residual extraction and direct oblimin rotation (gamma = 0). Interpretation was based on the pattern matrix. A primary loading of at least 0.40 was preferred. A secondary loading of at least 0.30 or a difference below 0.20 between the two largest loadings was treated as potential cross-loading. Item deletion was based jointly on loadings, communalities, item content, and the effect on the factor solution.

For CFA, ordinal indicators were analyzed using WLSMV with theta parameterization and standardized latent variances. Model-specific fit was evaluated using scaled chi-square, CFI, TLI, RMSEA with 90% confidence intervals, and SRMR. The nested two- and three-factor models were compared with a Satorra-corrected difference test. Model-based omega, average variance extracted (AVE), the Fornell-Larcker criterion, HTMT2 based on polychoric correlations, and latent-factor correlations were used to evaluate reliability and validity.

The EFA subsample contained no missing COM-B Item responses, and all COM-B Items used the full response range. Parallel analysis favored two factors. COM-B Item 7 showed no salient loading and very low communality in the competing EFA solutions and was removed. The two-factor solution without COM-B Item 7 remained stable, whereas the three-factor solution had lower residuals but retained a cross-loading of COM-B Item 3 on Capability and Opportunity.

In the independent CFA subsample, the three-factor model showed the best relative fit and fitted significantly better than the two-factor model. However, RMSEA remained elevated and the latent correlation between Capability and Opportunity was high. The results therefore supported use of the three theory-defined domain indicators with explicit qualification regarding the limited empirical separation of Capability and Opportunity.

Supplementary Table S1. COM-B Item wording, theoretical domain, and final retention decision

Item	Theoretical domain	Item wording or meaning	Decision
Item 1	Capability	I know the schedule of mandatory and recommended vaccinations.	Retained
Item 2	Capability	I can recognize false information about vaccination.	Retained
Item 3	Capability	I know where I can get vaccinated.	Retained
Item 4	Capability	I know the difference between mandatory and recommended vaccinations.	Retained
Item 5	Opportunity	Access to vaccination is easy where I live.	Retained
Item 6	Opportunity	I am able to consult a physician about vaccination.	Retained
Item 7	Opportunity	Vaccination is encouraged at my workplace or school.	Removed
Item 8	Motivation	People close to me view vaccination positively.	Retained
Item 9	Motivation	Vaccination is an effective way to protect public health.	Retained

Item	Theoretical domain	Item wording or meaning	Decision
Item 10	Motivation	I will get vaccinated to protect others.	Retained
Item 11	Motivation	I trust the institutions that recommend vaccination.	Retained
Item 12_R	Motivation	Reverse-scored item concerning fear of post-vaccination complications.	Retained

Note. COM-B Item 7 was excluded after EFA. The final regression indicators were Capability = mean (COM-B Items 1–4), Opportunity = mean (COM-B Items 5–6), and Motivation = mean (COM-B Items 8–11 and reverse-scored COM-B Item 12).

Supplementary Table S2. Preliminary item diagnostics and factorability

Item	Mean	SD	Median	Skewness	Floor, %	Ceiling, %	MSA
Item 1	3.007	1.203	3	-0.134	12.7	9.3	0.934
Item 2	3.499	0.932	4	-0.571	3.5	11.2	0.887
Item 3	4.034	0.837	4	-1.549	2.9	25.1	0.899
Item 4	3.942	0.861	4	-1.235	2.4	22.4	0.874
Item 5	3.899	0.862	4	-0.966	1.9	22.1	0.858
Item 6	3.991	0.828	4	-1.285	2.1	23.7	0.858
Item 7	2.970	0.982	3	-0.152	8.3	4.9	0.835
Item 8	3.230	1.027	3	-0.346	6.1	8.3	0.926
Item 9	3.793	0.992	4	-0.890	3.6	23.2	0.884
Item 10	3.503	1.090	4	-0.596	6.1	16.9	0.847
Item 11	3.246	1.150	3	-0.359	9.1	12.5	0.870
Item 12_R	2.650	1.120	2	0.294	14.9	4.9	0.881

Note. EFA subsample n = 1,500. Overall KMO = 0.879; item-level MSA = 0.835–0.934; determinant = 0.001333; smallest eigenvalue = 0.149; Bartlett's $\chi^2(66) = 9891.33$, $p < 0.001$; polychoric correlations ranged from 0.065 to 0.823.

Supplementary Table S3. Parallel analysis

Component	Observed eigenvalue	Random mean	Random 95th percentile	Decision
1	5.351	1.168	1.207	Retain
2	1.826	1.124	1.156	Retain
3	1.004	1.091	1.118	Do not retain
4	0.882	1.064	1.086	Do not retain
5	0.622	1.038	1.058	Do not retain
6	0.557	1.011	1.032	Do not retain
7	0.427	0.986	1.006	Do not retain
8	0.401	0.960	0.979	Do not retain
9	0.294	0.935	0.953	Do not retain
10	0.256	0.907	0.930	Do not retain
11	0.232	0.877	0.901	Do not retain
12	0.149	0.840	0.869	Do not retain

Note. Parallel analysis was based on 200 independently permuted datasets preserving the marginal distribution of each item.

Supplementary Table S4. Summary of competing EFA solutions

Item set	Factors	RMSR	Maximum |residual|	Interpretation
All 12	1	0.126	0.318	General factor; Item 7 very weak
All 12	2	0.043	0.140	Capability-Opportunity versus Motivation; Item 7 weak
All 12	3	0.028	0.097	Capability, Opportunity, and Motivation; Item 3 cross-loaded; Item 7 weak
Without COM-B Item 7	2	0.041	0.143	Stable two-factor solution
Without COM-B Item 7	3	0.020	0.047	Stable three-factor solution; Item 3 still cross-loaded

Supplementary Table S5. EFA pattern matrix and communalities before removal of COM-B Item 7: initial 12-item two-factor solution

Item	F1 Capability-Opportunity	F2 Motivation	Communality
Item 1	0.430	0.193	0.308
Item 2	0.428	0.123	0.253
Item 3	0.846	0.005	0.720
Item 4	0.813	-0.046	0.625
Item 5	0.795	-0.025	0.613
Item 6	0.729	0.058	0.579
Item 7	0.097	0.207	0.073
Item 8	0.021	0.737	0.560
Item 9	0.082	0.814	0.738
Item 10	0.009	0.881	0.784
Item 11	-0.021	0.910	0.808
Item 12_R	-0.123	0.655	0.361

Note. Factor correlation = 0.514.

Supplementary Table S6. EFA pattern matrix and communalities before removal of COM-B Item 7: initial 12-item three-factor solution

Item	F1 Capability	F2 Opportunity	F3 Motivation	Communality
Item 1	0.403	0.078	0.200	0.330
Item 2	0.708	-0.180	0.101	0.441
Item 3	0.526	0.385	0.041	0.700
Item 4	0.669	0.246	-0.035	0.677
Item 5	0.159	0.713	0.015	0.680
Item 6	-0.020	0.857	0.088	0.784
Item 7	-0.046	0.147	0.217	0.081
Item 8	-0.011	0.039	0.737	0.561
Item 9	0.010	0.084	0.817	0.740
Item 10	0.021	-0.002	0.876	0.783
Item 11	-0.031	0.016	0.906	0.809

Item	F1 Capability	F2 Opportunity	F3 Motivation	Communality
Item 12_R	0.069	-0.189	0.641	0.374

Note. Factor correlations: Capability–Opportunity = 0.595; Capability–Motivation = 0.441; Opportunity–Motivation = 0.416. COM-B Item 3 met the prespecified cross-loading criterion. COM-B Item 7 had no salient loading.

Supplementary Table S7. EFA pattern matrix and communalities after removal of COM-B Item 7: two-factor solution

Item	F1 Capability-Opportunity	F2 Motivation	Communality
Item 1	0.430	0.188	0.303
Item 2	0.429	0.124	0.254
Item 3	0.847	0.006	0.723
Item 4	0.815	-0.043	0.630
Item 5	0.794	-0.026	0.610
Item 6	0.729	0.056	0.576
Item 8	0.024	0.727	0.547
Item 9	0.083	0.817	0.743
Item 10	0.009	0.882	0.786
Item 11	-0.021	0.910	0.809
Item 12_R	-0.124	0.662	0.369

Note. Factor correlation = 0.514.

Supplementary Table S8. EFA pattern matrix and communalities after removal of COM-B Item 7: three-factor solution

Item	F1 Capability	F2 Opportunity	F3 Motivation	Communality
Item 1	0.427	0.060	0.190	0.333
Item 2	0.708	-0.174	0.099	0.443
Item 3	0.517	0.398	0.041	0.701
Item 4	0.648	0.267	-0.032	0.671
Item 5	0.163	0.708	0.013	0.673
Item 6	-0.029	0.869	0.086	0.793
Item 8	0.015	0.018	0.724	0.546
Item 9	-0.001	0.093	0.822	0.748
Item 10	0.019	-0.000	0.878	0.785
Item 11	-0.032	0.016	0.907	0.811
Item 12_R	0.056	-0.177	0.647	0.377

Note. Factor correlations: Capability–Opportunity = 0.589; Capability–Motivation = 0.439; Opportunity–Motivation = 0.418.

Supplementary Table S9. Fit of the competing confirmatory factor models in the independent CFA subsample - model structure and test statistics

Model	Structure	Scaled χ^2	df	p
One-factor	All 11 COM-B Items -> general factor	3359.48	44	<0.001
Two-factor	COM-B Items 1–6 → Capability–Opportunity; COM-B Items 8–11 and Item 12_R → Motivation	619.15	43	<0.001

Model	Structure	Scaled χ^2	df	p
Three-factor	COM-B Items 1–4 → Capability; COM-B Items 5–6 → Opportunity; COM-B Items 8–11 and Item 12_R → Motivation	523.23	41	<0.001

Supplementary Table S10. Fit of the competing confirmatory factor models in the independent CFA subsample - fit indices

Model	CFI	TLI	RMSEA	90% CI	SRMR
One-factor	0.871	0.839	0.224	0.218–0.231	0.153
Two-factor	0.978	0.971	0.095	0.088–0.101	0.053
Three-factor	0.981	0.975	0.089	0.082–0.095	0.048

Note. n = 1,500. Model-specific chi-square values are scaled statistics. The corrected comparison of the three-factor and two-factor models was significant, scaled $\Delta\chi^2(2) = 76.05$, $p < 0.001$. *All models converged and showed no negative variance estimates.*

Supplementary Table S11. Standardized CFA loadings and domain reliability in the final measurement model - fully standardized item loadings across the competing CFA models

Item	Final domain	One-factor loading	Two-factor loading	Three-factor loading
Item 1	Capability	0.477	Capability-Opportunity: 0.564	Capability: 0.576
Item 2	Capability	0.428	Capability-Opportunity: 0.540	Capability: 0.549
Item 3	Capability	0.723	Capability-Opportunity: 0.834	Capability: 0.861
Item 4	Capability	0.674	Capability-Opportunity: 0.780	Capability: 0.796
Item 5	Opportunity	0.657	Capability-Opportunity: 0.760	Opportunity: 0.821
Item 6	Opportunity	0.659	Capability-Opportunity: 0.765	Opportunity: 0.822
Item 8	Motivation	0.674	Motivation: 0.726	Motivation: 0.726
Item 9	Motivation	0.857	Motivation: 0.892	Motivation: 0.892
Item 10	Motivation	0.884	Motivation: 0.906	Motivation: 0.906
Item 11	Motivation	0.875	Motivation: 0.907	Motivation: 0.907
Item 12_R	Motivation	0.591	Motivation: 0.649	Motivation: 0.649

Supplementary Table S12. Standardized CFA loadings and domain reliability in the final measurement model - composite reliability and convergent validity of the three-factor model

Domain	Item s	Loading range	Omega	AVE	Square root of AVE	Qualification
Capability	Item 1-Item 4	0.549–0.861	0.717	0.580	0.762	Item 3 cross-loaded in EFA; retained on substantive and theoretical grounds.
Opportunity	Item 5-Item 6	0.821–0.822	0.730	0.675	0.822	Two-item domain; interpret cautiously.
Motivation	Item 8-Item 11, Item 12_R	0.649–0.907	0.889	0.749	0.866	Strongest and most clearly defined domain.

Supplementary Table S13. Latent correlations and discriminant-validity indicators for the three-factor CFA model

Domain pair	Latent r	95% CI	Square root of AVE	Fornell-Larcker criterion	HTMT2	HTMT2 < 0.85
Capability-Opportunity	0.840	0.815–0.865	0.762 / 0.822	Not met	0.789	Yes
Capability-Motivation	0.505	0.462–0.547	0.762 / 0.866	Met	0.489	Yes
Opportunity-Motivation	0.445	0.399–0.491	0.822 / 0.866	Met	0.405	Yes

Note. Evidence for discriminant validity between Capability and Opportunity was mixed: the Fornell-Larcker criterion was not met, although HTMT2 remained below 0.85.

Logistic regression model diagnostics

Both hierarchical logistic regression analyses used all 3,000 participants and had complete outcome and predictor data. Diagnostic checks addressed sparse cells, multicollinearity, linearity in the logit, nonlinear specification of vaccine hesitancy, convergence, residuals, leverage, and influence. No observation was excluded on the basis of these diagnostics.

Supplementary Table S14. Diagnostic assessment and final-model performance

Diagnostic or model characteristic	COVID-19 vaccination intention	Influenza vaccination intention
Analytical sample	N = 3,000; complete data	N = 3,000; complete data
Outcome events	547 (18.2%)	944 (31.5%)
Approximate events per estimated parameter	11.4	19.3
Minimum outcome-by-political-preference cell count	19	34
Smallest level of any categorical predictor	97 participants	97 participants
Tolerance range	0.246–0.867	0.246–0.867
VIF range	1.153–4.071	1.153–4.071
Linearity in the logit	No continuous predictor violated the assumption	Only vaccine hesitancy violated the assumption: Box-Tidwell $p = 0.000175$; Bonferroni threshold $p < 0.0083$
Nonlinear vaccine-hesitancy specification	Not required	Centered linear and squared terms retained; incremental improvement beyond the linear term: $\Delta\chi^2(1) = 13.19$, $p < 0.001$
Convergence	Converged after 6 iterations	Converged after 6 iterations
Influence diagnostics	No observation had Cook's distance > 1; no observation required exclusion	No observation had Cook's distance > 1; one case had Cook's distance = 0.816 and leverage = 0.00124; no observation required exclusion
Final -2 log likelihood	2037.600	2755.500
Final Cox-Snell R-squared	0.237	0.279
Final Nagelkerke R-squared	0.387	0.392
Hosmer-Lemeshow test	$\chi^2(8) = 11.29$, $p = 0.186$	$\chi^2(8) = 6.46$, $p = 0.596$
AUC (95% CI)	0.857 (0.842–0.873)	0.833 (0.818–0.848)

Note. The VIF and tolerance ranges refer to an auxiliary linear model that included the uncentered PL-aVHS mean score; the squared term used in the final influenza model was not included in this diagnostic. AUC values represent apparent discrimination in the estimation sample. The influenza Cox–Snell R^2 value was calculated from the reported model χ^2 and N using the standard Cox–Snell formula.

Post hoc analysis of the COM-B Opportunity coefficient

In the primary hierarchical logistic regression models, the COM-B Opportunity indicator showed an inverse coefficient after simultaneous adjustment for Capability and Motivation, although higher Opportunity was positively associated with both vaccination-intention outcomes when examined without the other COM-B domains. A targeted post hoc analysis was conducted to identify the model specification at which the coefficient changed direction.

Seven specifications were estimated separately for COVID-19 and influenza vaccination intentions: Opportunity alone; Opportunity adjusted for all variables entered in Blocks 1 and 2; Opportunity with Capability; Opportunity with Motivation; Opportunity with Capability and Motivation; the complete model containing Blocks 1 and 2 and all three COM-B indicators before vaccine hesitancy; and the final model including vaccine hesitancy. The final influenza model included the centered linear and squared vaccine-hesitancy terms.

Opportunity was positively associated with both outcomes when entered alone and remained positive after adjustment for Blocks 1 and 2. Adjustment for Capability attenuated both coefficients to nonsignificance. With Opportunity and Motivation entered together, the coefficient became negative for COVID-19 but remained close to zero for influenza. With all three COM-B domains entered simultaneously, Opportunity was inverse for both outcomes. The inverse coefficient was already present before vaccine hesitancy was added.

Supplementary Table S15. Sequence of the Opportunity coefficient across logistic regression model specifications

Model specification	COVID-19 B	COVID-19 OR	COVID-19 p	Influenza B	Influenza OR	Influenza p
1. Opportunity only	0.482	1.62	<0.001	0.510	1.66	<0.001
2. Opportunity + Blocks 1–2 covariates	0.285	1.33	<0.001	0.328	1.39	<0.001
3. Opportunity + Capability	0.109	1.12	0.179	0.085	1.09	0.207
4. Opportunity + Motivation	-0.158	0.85	0.047	0.023	1.02	0.724
5. Opportunity + Capability + Motivation	-0.225	0.80	0.014	-0.181	0.83	0.017
6. Blocks 1–2 covariates + Capability + Opportunity + Motivation	-0.300	0.74	0.002	-0.269	0.76	<0.001
7. Model 6 + vaccine hesitancy	-0.325	0.72	0.001	-0.311	0.73	<0.001

Note. Blocks 1–2 comprised sociodemographic, health-related, health-service-use, health-literacy, political, religious, social-media-use, and conspiracy-mentality variables. Models 3–5 were targeted COM-B-only specifications. In Model 7, vaccine hesitancy was entered as a centered linear term for COVID-19 and as centered linear and squared terms for influenza. In the final models, the 95% confidence intervals for the Opportunity OR were 0.59–0.88 for COVID-19 and 0.62–0.86 for influenza.

The sequence indicates that the inverse Opportunity coefficient was not present in the unadjusted or background-adjusted models. For COVID-19, the sign reversal appeared when Opportunity was considered jointly with Motivation; for influenza, it appeared after Capability and Motivation were included together. Vaccine hesitancy modestly strengthened but did not initiate the reversal.

Sensitivity analysis using a Motivation indicator excluding COM-B Item 10

The primary Motivation indicator was calculated as the unweighted mean of COM-B Items 8–11 and reverse-scored COM-B Item 12. Because COM-B Item 10 expressed willingness to be vaccinated to protect others and was therefore conceptually close to the vaccination-intention outcomes, both hierarchical models were re-estimated using an alternative Motivation indicator calculated as the mean of COM-B Items 8, 9, and 11 and reverse-scored COM-B Item 12. All remaining model specifications were unchanged, including the nonlinear vaccine-hesitancy specification in the influenza model.

Excluding COM-B Item 10 reduced the incremental contribution of the COM-B block from $\Delta\chi^2(3)$ = 339.19 to 305.53 for COVID-19 vaccination intention and from $\Delta\chi^2(3)$ = 428.84 to 376.48 for influenza vaccination intention. Final Nagelkerke R^2 decreased from 0.387 to 0.381 for COVID-19 and from 0.392 to 0.388 for influenza. The directions and statistical significance of Capability, Opportunity, Motivation, and vaccine hesitancy were retained.

Supplementary Table S16. Comparison of model fit between the primary and COM-B Item 10-excluded sensitivity models: incremental model fit

Outcome	Specification	COM-B block $\Delta\chi^2$ (df)	Nagelkerke R^2 after COM-B block	Final block $\Delta\chi^2$ (df)
COVID-19	Primary Motivation	339.19 (3)	0.371	37.32 (1)
COVID-19	Motivation without COM-B Item 10	305.53 (3)	0.357	56.92 (1)
Influenza	Primary Motivation	428.84 (3)	0.354	108.70 (2)
Influenza	Motivation without COM-B Item 10	376.48 (3)	0.336	150.53 (2)

Supplementary Table S17. Comparison of model fit between the primary and COM-B Item 10-excluded sensitivity models: final-model fit

Outcome	Specification	Final 2LL	Final Nagelkerke R^2	Hosmer-Lemeshow p
COVID-19	Primary Motivation	2037.60	0.387	0.186
COVID-19	Motivation without COM-B Item 10	2051.66	0.381	0.562
Influenza	Primary Motivation	2755.50	0.392	0.596
Influenza	Motivation without COM-B Item 10	2766.03	0.388	0.420

Note. The final block contained centered vaccine hesitancy in the COVID-19 model and centered linear plus squared vaccine-hesitancy terms in the influenza model. All omnibus block tests were significant at $p < 0.001$. Differences in -2LL between primary and sensitivity models are descriptive because the models used different composite versions of Motivation.

Supplementary Table S18. Key coefficients in the primary and COM-B Item 10-excluded models for COVID-19 vaccination intention

Predictor	Model	B	SE	OR	95% CI for OR	p
Capability	Primary	0.271	0.112	1.31	1.05–1.63	0.016
Capability	Without COM-B Item 10	0.297	0.112	1.35	1.08–1.68	0.008
Opportunity	Primary	-0.325	0.100	0.72	0.59–0.88	0.001

Predictor	Model	B	SE	OR	95% CI for OR	p
Opportunity	Without COM-B Item 10	-0.310	0.099	0.73	0.60–0.89	0.002
Motivation	Primary	0.970	0.145	2.64	1.99–3.51	<0.001
Motivation	Without COM-B Item 10	0.755	0.134	2.13	1.64–2.76	<0.001
Vaccine hesitancy (centered)	Primary	-0.970	0.162	0.38	0.28–0.52	<0.001
Vaccine hesitancy (centered)	Without COM-B Item 10	-1.147	0.156	0.32	0.23–0.43	<0.001

Note. ORs are adjusted for all variables included in the corresponding final model. The sensitivity model differed from the primary model only in the construction of Motivation.

Supplementary Table S19. Key coefficients in the primary and COM-B Item 10-excluded models for influenza vaccination intention

Predictor	Model	B	SE	OR	95% CI for OR	p
Capability	Primary	0.555	0.094	1.74	1.452.10	<0.001
Capability	Without COM-B Item 10	0.577	0.094	1.78	1.482.14	<0.001
Opportunity	Primary	-0.311	0.084	0.73	0.620.86	<0.001
Opportunity	Without COM-B Item 10	-0.302	0.083	0.74	0.630.87	<0.001
Motivation	Primary	0.442	0.112	1.56	1.251.94	<0.001
Motivation	Without COM-B Item 10	0.240	0.105	1.27	1.031.56	0.023
Vaccine hesitancy (centered linear term)	Primary	-1.494	0.158	0.22	0.160.31	<0.001
Vaccine hesitancy (centered linear term)	Without COM-B Item 10	-1.675	0.154	0.19	0.140.25	<0.001
Vaccine hesitancy squared	Primary	-0.393	0.113	0.67	0.540.84	0.001
Vaccine hesitancy squared	Without COM-B Item 10	-0.409	0.113	0.66	0.530.83	<0.001

Note. The exponentiated linear and squared vaccine-hesitancy coefficients must be interpreted jointly because vaccine hesitancy was modeled as a polynomial. ORs are adjusted for all variables included in the corresponding final model.

Interpretation of the sensitivity analysis

Excluding COM-B Item 10 attenuated the Motivation coefficient, particularly in the influenza model, but did not change the direction or statistical significance of the focal associations. The small reductions in final Nagelkerke R^2 and preservation of the Capability, Opportunity, Motivation, and vaccine-hesitancy coefficients support the robustness of the primary findings. The primary models were retained because they used the prespecified Motivation indicator and had slightly better overall fit.

frontiers

Strobe checklist

STROBE Statement—Completed checklist for a cross-sectional study

Manuscript: Shared and vaccine-specific factors associated with COVID-19 and influenza vaccination intentions among adults in Poland: a COM-B-informed cross-sectional study

Supplementary Table S20. Strobe checklist

Section/topic	Item No.	STROBE recommendation	Where reported in the manuscript / action required
**Title and abstract**			
Title and abstract	**1(a)**	Indicate the study's design with a commonly used term in the title or the abstract.	Reported: p. 1, title; Abstract, pp. 1–2, lines 12–40. The title explicitly identifies the study as cross-sectional, and the Abstract states the cross-sectional CAWI design.
Title and abstract	**1(b)**	Provide in the abstract an informative and balanced summary of what was done and what was found.	Reported: Abstract, pp. 1–2, lines 12–40. It summarizes the aim, CAWI design, N = 3,000, measures, independent EFA/CFA subsamples, hierarchical regression, principal associations, model fit, and conclusions.
**Introduction**			
Background/rationale	**2**	Explain the scientific background and rationale for the investigation being reported.	Reported: Introduction, pp. 2–4, lines 43–158. The rationale covers vaccine-specific intentions, sociodemographic and health context, health literacy, social media, conspiracy mentality, 5C/7C, COM-B, adult vaccine hesitancy, and the rationale for comparing COVID-19 and influenza intentions.
Objectives	**3**	State specific objectives, including any prespecified hypotheses.	Reported: final paragraph of the Introduction, p. 4, lines 159–167. The objectives specify shared and vaccine-specific associations, psychometric evaluation of the COM-B-informed item set, the incremental contribution of vaccine hesitancy, and post hoc/sensitivity analyses. No directional hypotheses were prespecified.
**Methods**			
Study design	**4**	Present key elements of study design early in the paper.	Reported: title and Abstract; Material and methods, p. 5, lines 169–204. The study is identified as a cross-sectional CAWI survey using a non-probability quota sample.
Setting	**5**	Describe the setting, locations, and relevant dates, including periods of recruitment, exposure, follow-up, and data collection.	Reported: p. 5, lines 170–177. Data were collected in Poland from May 20 to May 26, 2026, through the IQS online research panel. Follow-up was not applicable.
Participants	**6(a)**	Give the eligibility criteria, and the sources and methods of selection of participants.	Reported: p. 5, lines 171–186. Eligible participants were IQS panel members aged 18–75 years who resided in Poland and provided electronic informed consent. Recruitment used prespecified quotas for age, sex, educational attainment, place of residence, and NUTS 1 macroregion; exclusion criteria and data-quality exclusions are also described.
Variables	**7**	Clearly define all outcomes, exposures, predictors, potential confounders, and effect modifiers. Give diagnostic criteria, if applicable.	Reported: Questionnaire and measure subsections, pp. 5–8, lines 205–306; Statistical analysis, pp. 8–10, lines 307–392; Tables 1, 3, and 4 (pp. 23–28). Outcomes and all predictors/covariates are defined. No effect modifiers were modeled.
Data sources/measurement	**8***	For each variable of interest, give sources of data and details of methods of assessment (measurement). Describe comparability of assessment methods if there is more than one group.	Reported: pp. 5–9, lines 205–348. The questionnaire, response options, score construction, item reversal, validity rules, categorical groupings, current-sample reliability, and COM-B domain-indicator construction are described. The same assessment methods were used for all respondents.
Bias	**9**	Describe any efforts to address potential sources of bias.	Reported across Methods and Limitations: quota controls, non-probability sampling, and unweighted analysis (p. 5, lines 173–177); attention checks, completion-time criterion, and straight-lining exclusions (p. 5, lines 178–186); rotation of selected scale items (p. 6, lines 211–216); independent EFA/CFA split (p. 8, lines 318–323); regression diagnostics (p. 9, lines 364–373; Supplementary Table S14); and limitations (pp. 16–17, lines 658–692).
Study size	**10**	Explain how the study size was arrived at.	Reported: p. 5, lines 187–196. The target N = 3,000 was selected pragmatically to support independent EFA/CFA subsamples of 1,500, multivariable regression, and assessment of less prevalent response categories and distribution tails. The nominal ±1.8 percentage-point benchmark is explicitly qualified as not being a design-based sampling error.
Quantitative variables	**11**	Explain how quantitative variables were handled in the analyses. If applicable, describe which groupings were chosen and why.	Reported: pp. 7–9, lines 253–363, and Tables 1, 3, and 4. Continuous scores, categorical groupings and boundaries, HLS-EU-Q6 validity thresholds, mean centering of AVHS, and the centered quadratic term for influenza are described.

frontiers

Section/topic	Item No.	STROBE recommendation	Where reported in the manuscript / action required
Statistical methods	**12(a)**	Describe all statistical methods, including those used to control for confounding.	Reported: Statistical analysis, pp. 8–10, lines 307–392. EFA, CFA, reliability/validity assessment, hierarchical logistic regression, forced-entry blocks, Box–Tidwell tests, multicollinearity, influence diagnostics, model fit, AUC, and adjusted ORs are described. Blocks 1–4 define the adjustment strategy.
Statistical methods	**12(b)**	Describe any methods used to examine subgroups and interactions.	No subgroup or interaction models were estimated. Targeted post hoc specifications examined the Opportunity coefficient (p. 10, lines 379–387; Supplementary Table S15).
Statistical methods	**12(c)**	Explain how missing data were addressed.	Reported: HLS-EU-Q6 scoring rules, p. 7, lines 253–265; Model diagnostics, p. 11, line 440. "Difficult to say/not applicable" responses were treated as nonvalid for HLS-EU-Q6 scoring, respondents with insufficient valid items were retained in an undetermined category, and the final regression analyses had complete outcome and predictor data for all 3,000 participants.
Statistical methods	**12(d)**	If applicable, describe analytical methods taking account of sampling strategy.	Reported: p. 5, lines 173–177. The study used a non-probability quota sample and all descriptive and inferential analyses were conducted using unweighted data.
Statistical methods	**12(e)**	Describe any sensitivity analyses.	Reported: p. 10, lines 388–392; Results, p. 12, lines 500–507; Supplementary Tables S16–S19. Both hierarchical models were re-estimated with a Motivation indicator excluding COM-B Item 10.
**Results**			
Participants	**13(a)**	Report numbers of individuals at each stage of study—e.g., numbers potentially eligible, examined for eligibility, confirmed eligible, included in the study, completing follow-up, and analyzed.	Reported: p. 5, lines 178–186. A total of 12,432 invitations were sent; 4,074 respondents entered the questionnaire; 1,074 cases were excluded; and 3,000 completed and approved interviews were included in the final dataset and analyzed. Follow-up was not applicable.
Participants	**13(b)**	Give reasons for non-participation at each stage.	Reported: p. 5, lines 178–186. Reasons included non-participation, declined consent, technical inability to continue, incomplete questionnaires, failed attention checks, completion in less than one-third of the median completion time, and substantial straight-line response patterns.
Participants	**13(c)**	Consider use of a flow diagram.	No flow diagram is included. Participant flow and reasons for exclusion are reported numerically in the Methods (p. 5, lines 178–186).
Descriptive data	**14(a)**	Give characteristics of study participants (e.g., demographic, clinical, social) and information on exposures and potential confounders.	Reported: Results, Sample characteristics, p. 10, lines 394–403; Table 1, pp. 23–24. Demographic, social, health-related, vaccination-intention, AVHS, conspiracy-mentality, and COM-B domain characteristics are presented.
Descriptive data	**14(b)**	Indicate number of participants with missing data for each variable of interest.	Reported: p. 11, line 440, and Supplementary Table S14. Both regression models included all 3,000 participants with complete outcome and predictor data. For HLS-EU-Q6, respondents without enough valid items were retained in an "undetermined" category (n = 490; Table 1, pp. 23–24).
Outcome data	**15***	Report numbers of outcome events or summary measures.	Reported: p. 10, lines 395–403, and Table 1, pp. 23–24. COVID-19 vaccination intention was reported by 547 participants (18.2%) and influenza vaccination intention by 944 participants (31.5%).
Main results	**16(a)**	Give unadjusted estimates and, if applicable, confounder-adjusted estimates and their precision (e.g., 95% confidence interval). Make clear which confounders were adjusted for and why they were included.	Partially reported: the fully adjusted estimates with 95% CIs are reported for COVID-19 (pp. 11–12, lines 461–470; Table 3, pp. 25–26) and influenza (p. 12, lines 471–489; Table 4, pp. 27–28). The adjustment set is defined by Blocks 1–4 in Methods (p. 9, lines 349–357) and Table 2 (p. 24). Unadjusted/sequential Opportunity estimates are reported in Supplementary Table S15, but unadjusted estimates are not systematically presented for every predictor.
Main results	**16(b)**	Report category boundaries when continuous variables were categorized.	Reported: Methods, pp. 7–8, lines 253–306, and Tables 1, 3, and 4. Boundaries for health literacy, settlement size, income, physician-service use, social-media use, and other grouped variables are stated; age was retained as a continuous variable.
Main results	**16(c)**	If relevant, consider translating estimates of relative risk into absolute risk for a meaningful time period.	Not applicable. The outcomes were cross-sectional binary intentions rather than incident events over a defined follow-up period; adjusted odds ratios were reported.
Other analyses	**17**	Report other analyses done—e.g., analyses of subgroups and interactions, and sensitivity analyses.	Reported: p. 12, lines 494–507; Supplementary Table S15 (post hoc Opportunity sequence) and Supplementary Tables S16–S19 (sensitivity analysis excluding COM-B Item 10). No subgroup or interaction analyses were conducted.
**Discussion**			
Key results	**18**	Summarize key results with reference to study objectives.	Reported: Discussion, Principal findings, p. 13, lines 509–523; Conclusions, p. 17, lines 693–703. Shared and vaccine-specific associations, COM-B domain results, the incremental contribution of vaccine hesitancy, and the nonlinear influenza finding are summarized.

Section/topic	Item No.	STROBE recommendation	Where reported in the manuscript / action required
Limitations	**19**	Discuss limitations of the study, taking into account sources of potential bias or imprecision. Discuss both direction and magnitude of any potential bias.	Reported: Strengths and limitations, pp. 16–17, lines 658–692. Limitations include the cross-sectional design, dichotomized intention outcomes, survey-specific COM-B indicators, limited Opportunity coverage, self-report/common-method effects, measurement granularity, country/time specificity, apparent AUC, and omitted determinants. Potential bias directions are discussed qualitatively where possible; exact magnitudes cannot be estimated.
Interpretation	**20**	Give a cautious overall interpretation of results considering objectives, limitations, multiplicity of analyses, results from similar studies, and other relevant evidence.	Reported: Discussion and Conclusions, pp. 13–17, lines 509–703. The Opportunity coefficient is interpreted cautiously as conditional/suppression-related, vaccine hesitancy is treated as incremental rather than causal, vaccine-specific patterns are compared with prior evidence, and limitations are incorporated into the overall interpretation.
Generalisability	**21**	Discuss the generalizability (external validity) of the study results.	Reported: p. 17, lines 683–692. The manuscript limits inference to Poland in 2026, notes the post-pandemic and vaccine-specific context, and states that the magnitude of associations may differ across seasons, policy settings, and populations; external validation is required before predictive use elsewhere.
**Other information**			
Funding	**22**	Give the source of funding and the role of the funders for the present study and, if applicable, for the original study on which the present article is based.	Reported: Funding, p. 18, lines 722–728. The study and publication were supported by internal research funding at Jagiellonian University Medical College, project N43/GBS/000002. The funder had no role in the study design, data collection, data analysis, interpretation of the results, preparation of the manuscript, or the decision to submit the article for publication.

*The original checklist asks that information be provided separately for exposed and unexposed groups. This qualification is not directly applicable because the present study did not define exposed and unexposed groups.

Source: Official STROBE checklist for cross-sectional studies, STROBE Initiative.
